# Supplementary material for: Recovering the pre-colonial population structure of Khoe-San descendant populations
Source: bioRxiv. 2025 Oct 7:2025.10.05.680541. Preprint. [Version 1] doi: 10.1101/2025.10.05.680541 (PMC12632568; doi:10.1101/2025.10.05.680541)
Supplement: Supplement 1 [file NIHPP2025.10.05.680541v1-supplement-1.pdf]

bioRxiv preprint doi: https://doi.org/10.1101/2025.10.05.680541; this version posted October 7, 2025. The copyright holder for this preprint (which was not certified by peer review) is the author/funder, who has granted bioRxiv a license to display the preprint in perpetuity. It is made available under aCC-BY 4.0 International license.

Supplemental Results: Table of Contents

Inferring Botswanan Populations 3

Supplemental Figure 1) *Identifying the Botswanan San using ADMIXTURE* 4

Supplemental Figure 2) *Differentiating Botswanan San Populations* 5

Supplemental Figure 3) *UMAP and HBDscan of reference and query populations* 6

Supplemental Figure 4) *PC1 through PC10* 7

Characterizing sampled Coloured populations 8

Supplemental Figure 5) *Identifying relatives' workflow* 8

Supplemental Figure 6) *Unsupervised ADMIXTURE* 9

Supplemental Figure 7) *Cross-validation error rate for ADMIXTURE* 10

Supplemental Figure 8) *Proportion of Khoe-San ancestry by distance from Cape Town* 11

Supplemental Figure 9) *Linear regression* 12

Local ancestry results 13

Supplemental Figure 10) *Local Ancestry and ancestry-specific MDS pipeline* 13

Supplemental Figure 11) *Local ancestry inference confusion matrix* 14

Supplemental Figure 12) *GNOMIX population-specific global ancestry average proportions.* 15

Supplemental Figure 13) *Correlation between GNOMIX global ancestry results and ADMIXTURE* 16

Supplemental Figure 14) *Track lengths* 17

Ancestry-specific MDS results 18

Supplemental Figure 15) *NKA asMDS results by population* 18

Supplemental Figure 16) *European and South Asian asMDS* 19

Supplemental Figure 17) *Zulu derived Khoe-San asMDS.* 20

Admixture Proportions 21

Supplemental Table 1) *Sample Size for each population included in ADMIXTURE* 21

Supplemental Table 2) *ADMIXTURE k=5* 22

Supplemental Table 3) *ADMIXTURE k=8* 23

Supplemental Table 4) *ADMIXTURE k=10* 24

Population Centroids and Euclidean Distances 25

Supplemental Figure 18) *Khoe-San derived population centroids* 26

Supplemental Figure 19) *Non-Khoe-San derived population centroids* 27

Supplemental Figure 20) *European derived centroids* 28

Supplemental Figure 21) *Other Asian derived centroids* 29

bioRxiv preprint doi: https://doi.org/10.1101/2025.10.05.680541; this version posted October 7, 2025. The copyright holder for this preprint (which was not certified by peer review) is the author/funder, who has granted bioRxiv a license to display the preprint in perpetuity. It is made available under aCC-BY 4.0 International license.

Supplemental Figure 22) *South Asian derived centroids* *30*

Supplemental Table 5) *Khoe-San* *31*

Supplemental Table 6) *Non-Khoe-San* *31*

Supplemental Table 7) *European* *32*

Supplemental Table 8) *South Asian* *32*

Supplemental Table 9) *Southeast and East Asian* *33*

SPRUCE **34**

Supplemental Figure 23) *Error rate plot showing* *34*

Supplemental Figure 24) *Scatterplot showing predicted migration plotted against observed migration* ... *35*

Supplemental Figure 25) *Error rate plot* *36*

Supplemental Figure 26) *Scatterplot showing predicted migration plotted against observed migration* ... *37*

Supplemental Figure 27) *SPRUCE Random forest* *38*

Supplemental Figure 28) *Scatterplot showing* *39*

References **41**

bioRxiv preprint doi: https://doi.org/10.1101/2025.10.05.680541; this version posted October 7, 2025. The copyright holder for this preprint (which was not certified by peer review) is the author/funder, who has granted bioRxiv a license to display the preprint in perpetuity. It is made available under aCC-BY 4.0 International license.

Inferring Botswanan Populations

We began by running an unsupervised ADMIXTURE inferring 2 to 7 population clusters (k) with the entirety of the Crawford et al. (2017) data (Supplemental Figure 1), along with the GBR, BEB, STU, GIH, KHV, IND, YRI, HMB, LWK, Zulu, Gumuz, Somali, Wolayta, Omoro, KHM, and Nama, and query populations. From there, we subset the Crawford et al. (2017) data for the 314 individuals with the highest Khoe-San ancestry proportion (Supplemental Figure 1A), as that is the reported number of San individuals sampled in the study,[1] and then re-ran ADMIXTURE k2 through k7 with those individuals (Supplemental Figure 1B).

We then filtered the Botswanan San, ≠Khomani, and Nama individuals with 85% or greater Khoe-San ancestry as inferred by ADMIXTURE k5 and plotted them on a PCA (Supplemental Figure 2A). We observe a previously reported cline among the Kalahari San populations, ≠Khomani, and Nama. The Botswanan San split into two clusters, one of which anchored a corner of PC2. From there, we were able to infer the cluster of Botswanan San individuals diving PC1 likely have greater genetic variation relative to the other high Khoe-San populations. This leads us to infer that cluster of individuals to be the !Xoo, who were reported to have a higher population effective size relative to the Ju|'haonsi.[2]

We continued to our KHS ancestry-specific MDS. We add ≠Khomani, Nama, and Botswanan San individuals who did not meet the 85% Khoe-San ancestry cut-off into the query. We project those haplotypes, along with all first- and second-degree relatives in the data set, onto an MDS with the 85% and greater KHS reference. Projecting these individuals minimizes the effect relatives and admixture can have on dimensionality reduction. We use the mclust[3] R package to read in MDS2 data for the Botswanan San, the dimension where the Botswanan San split into two clusters. This package is designed for complex clustering and classification using a Gaussian finite mixture, meaning it assumes the data is made up of several Gaussian distributions. We use it to model MDS2 distribution with two clusters and identify the mean and standard deviation of both clusters (Supplemental Figure 2B-D). We filtered our model results to include only individuals with a probability of 0.9 or higher of belonging to a cluster. Upon examining samples across both clusters, we found no overlap between individuals assigned to each cluster. Most individuals who did not meet the ≥0.9 probability threshold are those positioned between the two cluster peaks, i.e., more than one standard deviation away from the modeled mean. These 10 individuals were labeled as BSAN_UNK (Botswanan San, Unknown). The individuals within the cluster positioned on the right side of MDS2 were labeled !Xoo, as they represent the samples contributing most to the genetic variation in MDS1. This classification leaves the remaining cluster of individuals to be identified as Ju|'hoansi (Supplemental Figure 3).

In order to refine the clustering of the assigned BSAN_UNK individuals, we performed a dimensionality reduction with uniform manifold approximation and projection (UMAP)[4] followed by genetic clustering with Hierarchical Density-Based Spatial Clustering of Applications with Noise implementation, HDBSCAN($\hat{\varepsilon}$).[5] We used as input the first 100 principal components (PCs) obtained with SmartPCA program from EIGENSOFT 7.2.1[6] and applied the same approach described in Diaz-Papkovich et al 2023.[7] For visualization purposes, UMAP was run with 25 number of nearest neighbors, a minimum distance of 0.3, and a dimension reduction of 2 components. For clustering, UMAP parameters included 50 number of neighbors, minimum distance of 0.01 and 5 number of components. Next, HDBSCAN($\hat{\varepsilon}$) was used to extract clusters using an $\hat{\varepsilon}$ of 0.3 and a minimum number of points in a cluster of 50.

bioRxiv preprint doi: https://doi.org/10.1101/2025.10.05.680541; this version posted October 7, 2025. The copyright holder for this preprint (which was not certified by peer review) is the author/funder, who has granted bioRxiv a license to display the preprint in perpetuity. It is made available under aCC-BY 4.0 International license.

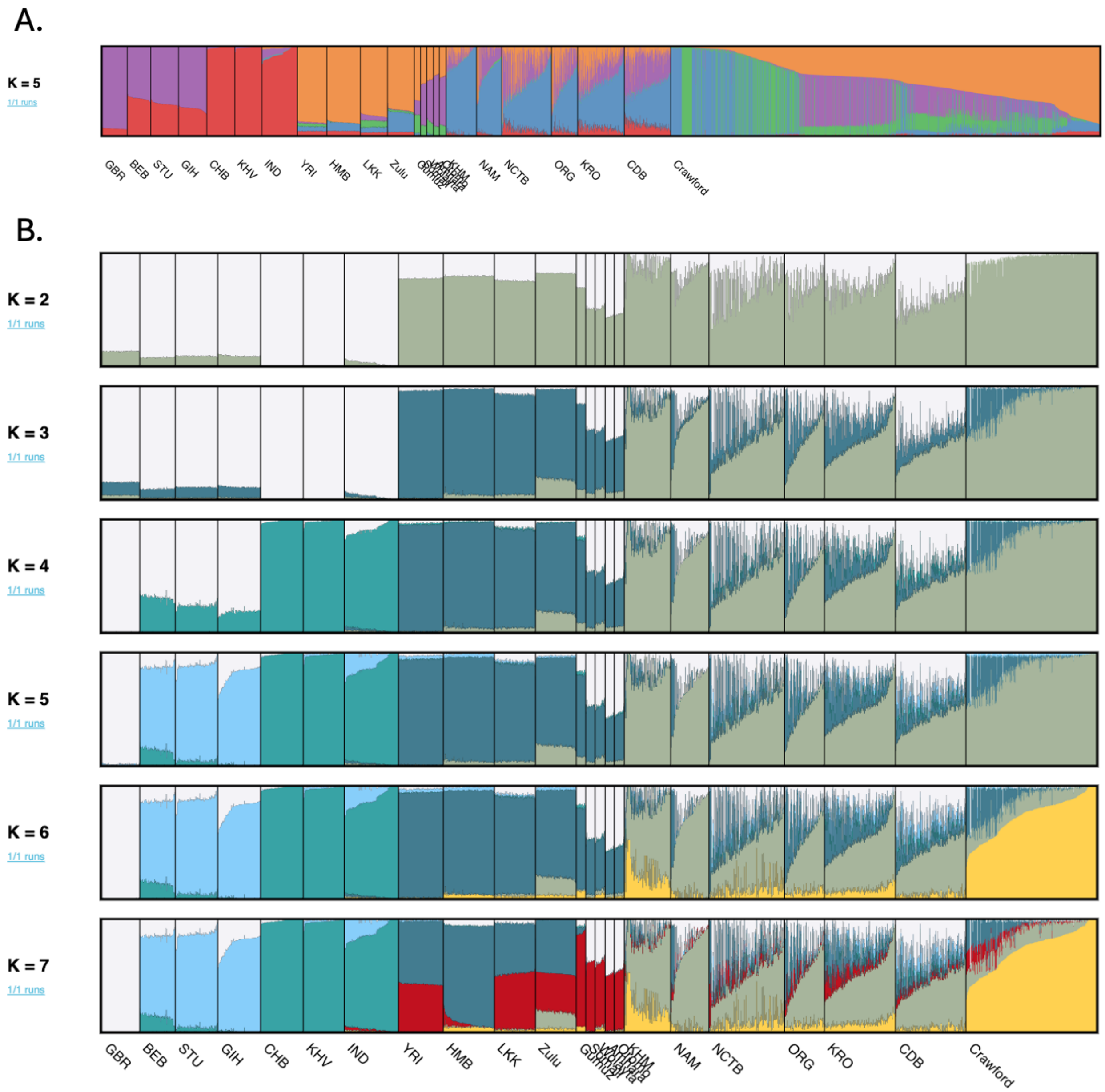

Supplemental Figure 1) Identifying the Botswanan San using ADMIXTURE. **A)** ADMIXTURE at k5 with the entire Crawford et al. (2017) data. **B)** ADMIXTURE k2 through k7 with only 314 Botswanan San with the highest Khoe-San ancestry as inferred by figure A's results.

bioRxiv preprint doi: https://doi.org/10.1101/2025.10.05.680541; this version posted October 7, 2025. The copyright holder for this preprint (which was not certified by peer review) is the author/funder, who has granted bioRxiv a license to display the preprint in perpetuity. It is made available under aCC-BY 4.0 International license.

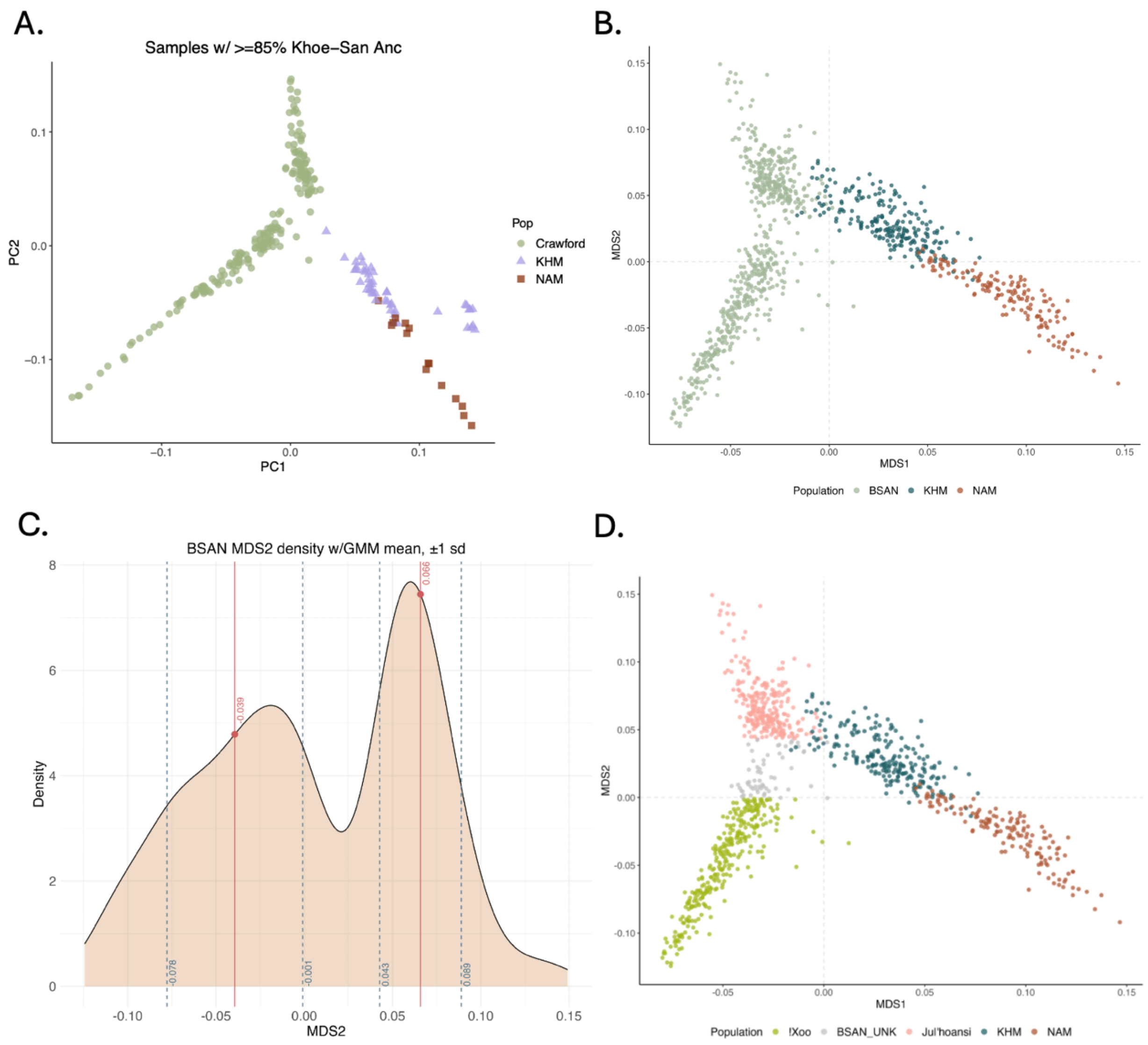

Supplemental Figure 2) Differentiating Botswanan San Populations. **A)** Individuals with greater than 85% Khoe-San ancestry as inferred by ADMIXTURE k5 from Supplemental Figure 1A. **B)** Khoe-San (KHS) ancestry-specific multidimensional scaling (MDS) 1 and 2 of KHS reference populations. Individuals with less than 85% Khoe-San ancestry were added to the local ancestry inference query and projected into the MDS space, along with first- and second-degree relatives. **C)** mclust Gaussian mixture model identified mean and 1 standard deviation of MDS2 Botswanan San density. Red line specified averages while dashed blue lines highlight the 1 standard deviation cut offs. **D)** Cluster/population assignment as inferred by the Gaussian mixture model from mclust R package and previously published population effective size data[2].

bioRxiv preprint doi: https://doi.org/10.1101/2025.10.05.680541; this version posted October 7, 2025. The copyright holder for this preprint (which was not certified by peer review) is the author/funder, who has granted bioRxiv a license to display the preprint in perpetuity. It is made available under aCC-BY 4.0 International license.

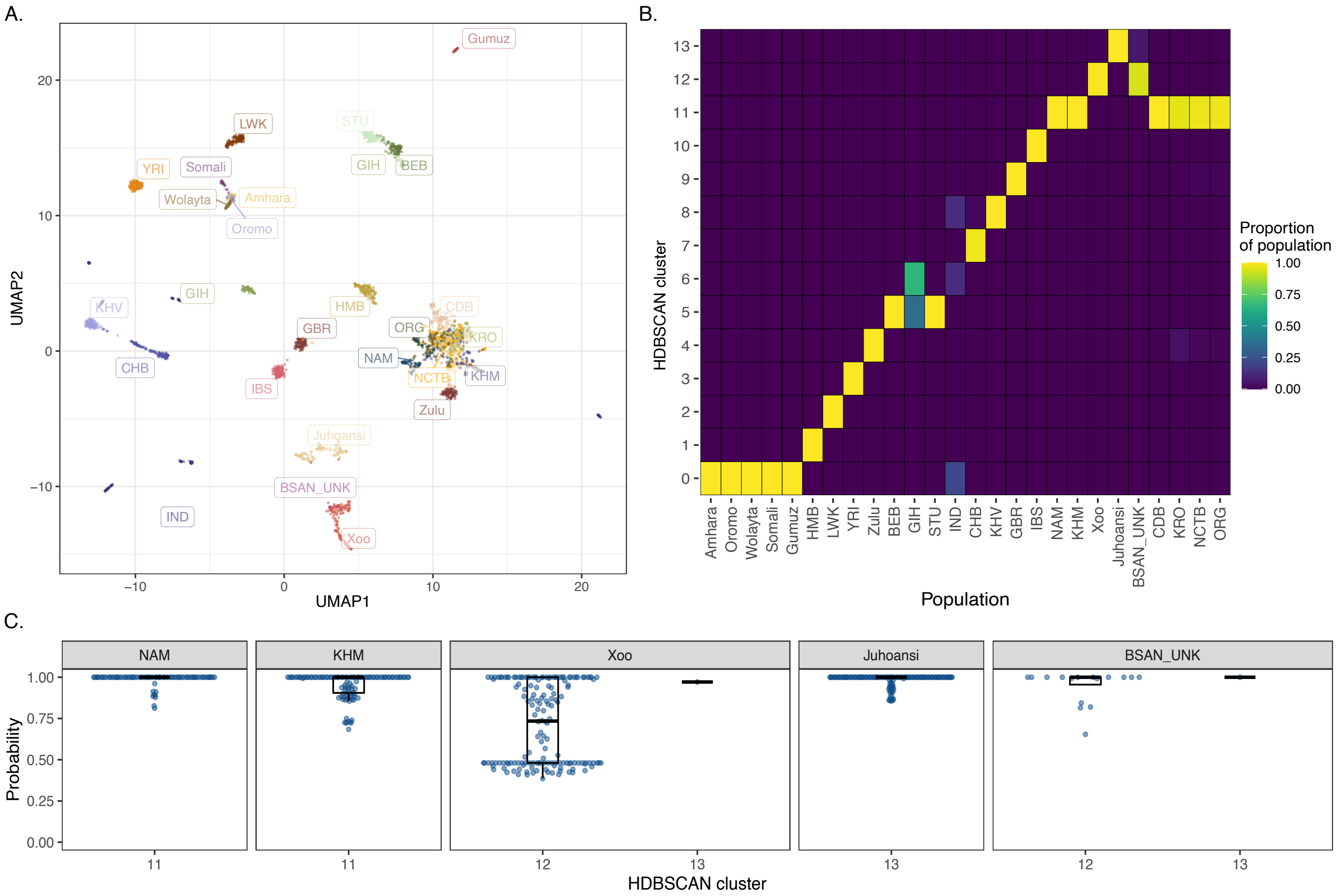

Supplemental Figure 3) UMAP and HBDscan of reference and query populations **A.** UMAP generated with 25 nearest neighbors, and minimum distance of 0.3. Labels refer to population labels. **B.** Proportion of individuals of each population contained within a given cluster inferred from HDBscan algorithm (see Methods for further details on the parameters used). **C.** Distribution probabilities per individual to be in the cluster inferred in B for each Khoe-San reference population.

bioRxiv preprint doi: https://doi.org/10.1101/2025.10.05.680541; this version posted October 7, 2025. The copyright holder for this preprint (which was not certified by peer review) is the author/funder, who has granted bioRxiv a license to display the preprint in perpetuity. It is made available under aCC-BY 4.0 International license.

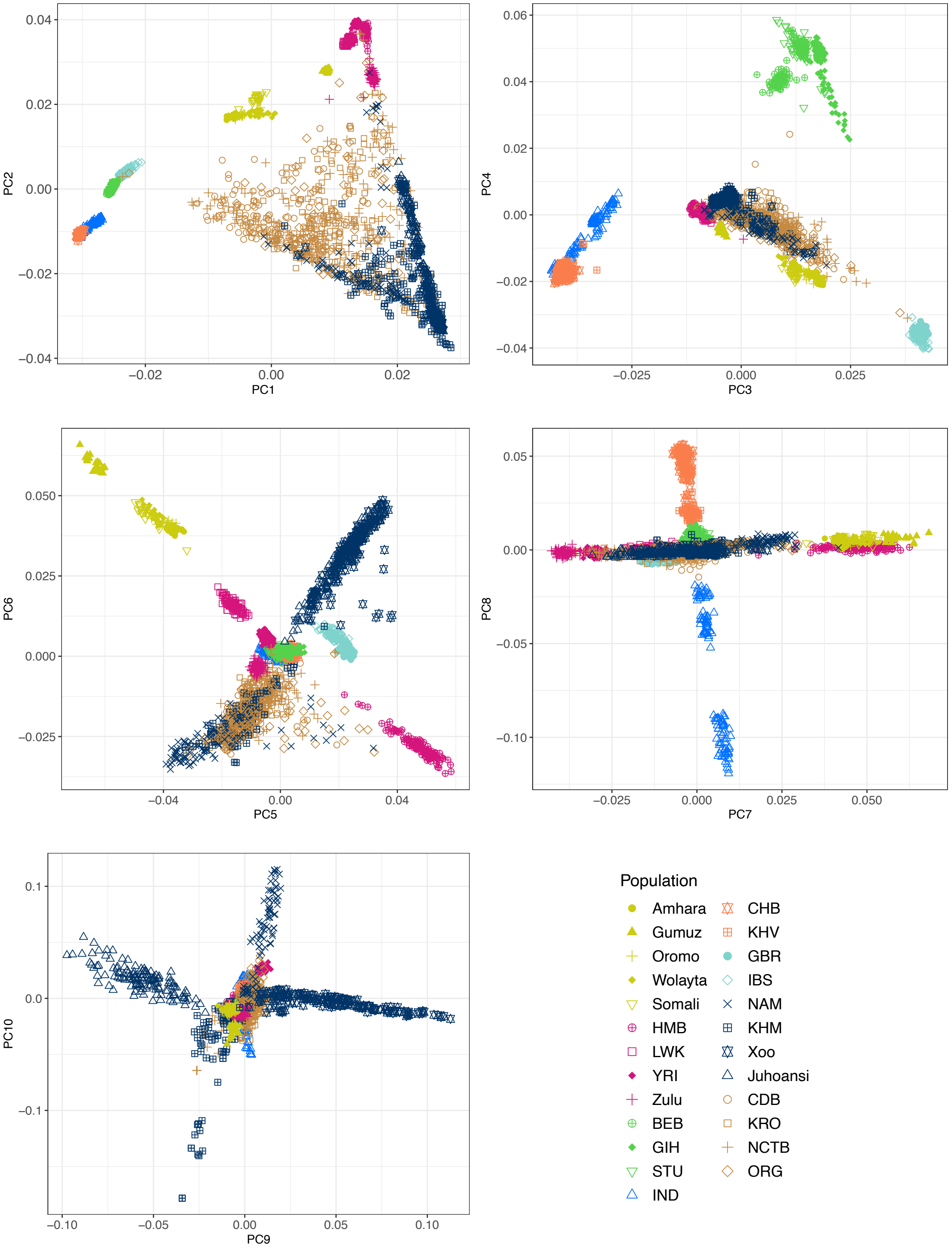

Supplemental Figure 4) PC1 through PC10. PCs 1 through 10 were generated using smartPCA and plotted using ggplot2 in R. Colors roughly represent broader ancestry, while shapes represent population.

bioRxiv preprint doi: https://doi.org/10.1101/2025.10.05.680541; this version posted October 7, 2025. The copyright holder for this preprint (which was not certified by peer review) is the author/funder, who has granted bioRxiv a license to display the preprint in perpetuity. It is made available under aCC-BY 4.0 International license.

Characterizing sampled Coloured populations

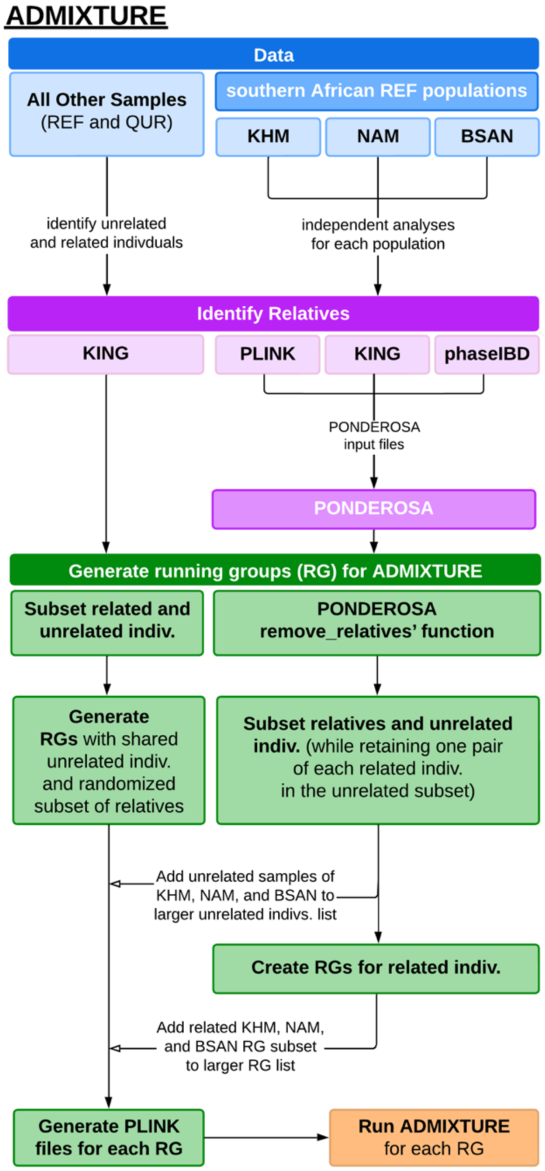

Supplemental Figure 5) Identifying relatives' workflow. Visual of applied workflow used to identify relatives and subset samples into running groups (RG) for ADMIXTURE. Each RG contained the same set of unrelated individuals but a different subset of related individuals. They were all subject to 10 iterations of ADMIXTURE, all with a different initial starting seed and cross-validated five times. PLINK LD pruning was completed prior to sub-setting samples, but after identifying relatives. PLINK LD pruning not depicted in the workflow.

bioRxiv preprint doi: https://doi.org/10.1101/2025.10.05.680541; this version posted October 7, 2025. The copyright holder for this preprint (which was not certified by peer review) is the author/funder, who has granted bioRxiv a license to display the preprint in perpetuity. It is made available under aCC-BY 4.0 International license.

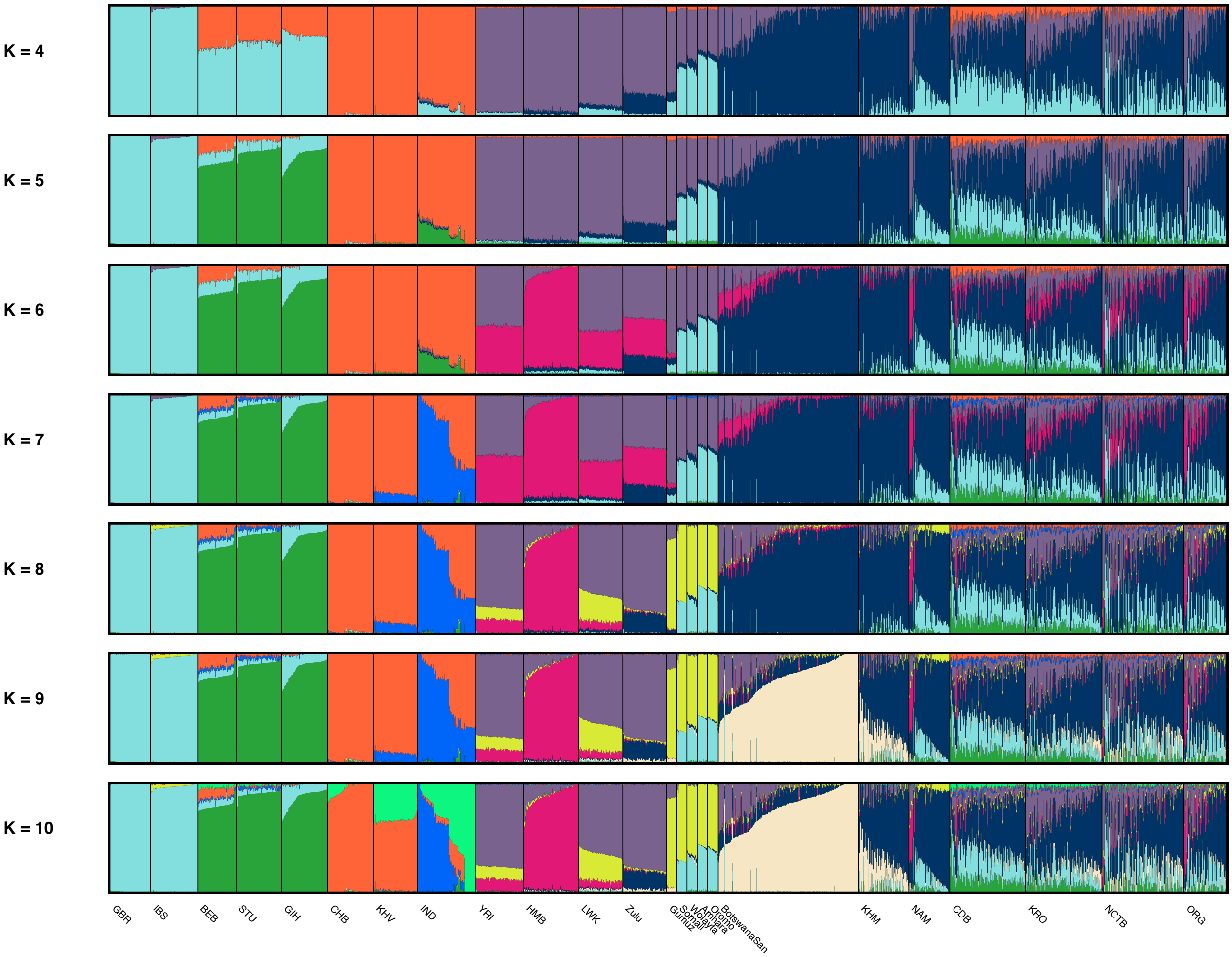

Supplemental Figure 6) Unsupervised ADMIXTURE run of *k*=4 through *k*=10 using final merged dataset of 2,505 individuals and 806,460 markers congruent H3Africa array.

bioRxiv preprint doi: https://doi.org/10.1101/2025.10.05.680541; this version posted October 7, 2025. The copyright holder for this preprint (which was not certified by peer review) is the author/funder, who has granted bioRxiv a license to display the preprint in perpetuity. It is made available under aCC-BY 4.0 International license.

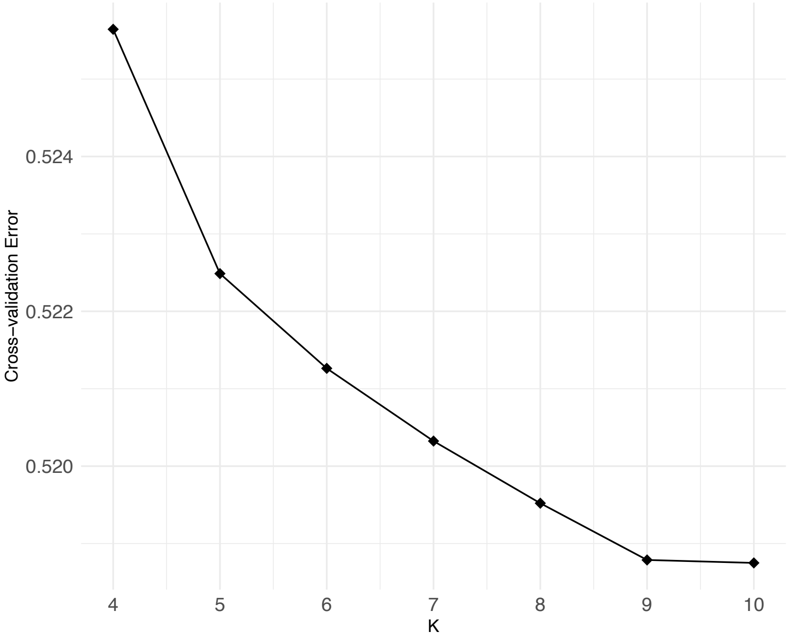

Supplemental Figure 7) Cross-validation error rate for ADMIXTURE visualized in Supplemental Figure 6.

bioRxiv preprint doi: https://doi.org/10.1101/2025.10.05.680541; this version posted October 7, 2025. The copyright holder for this preprint (which was not certified by peer review) is the author/funder, who has granted bioRxiv a license to display the preprint in perpetuity. It is made available under aCC-BY 4.0 International license.

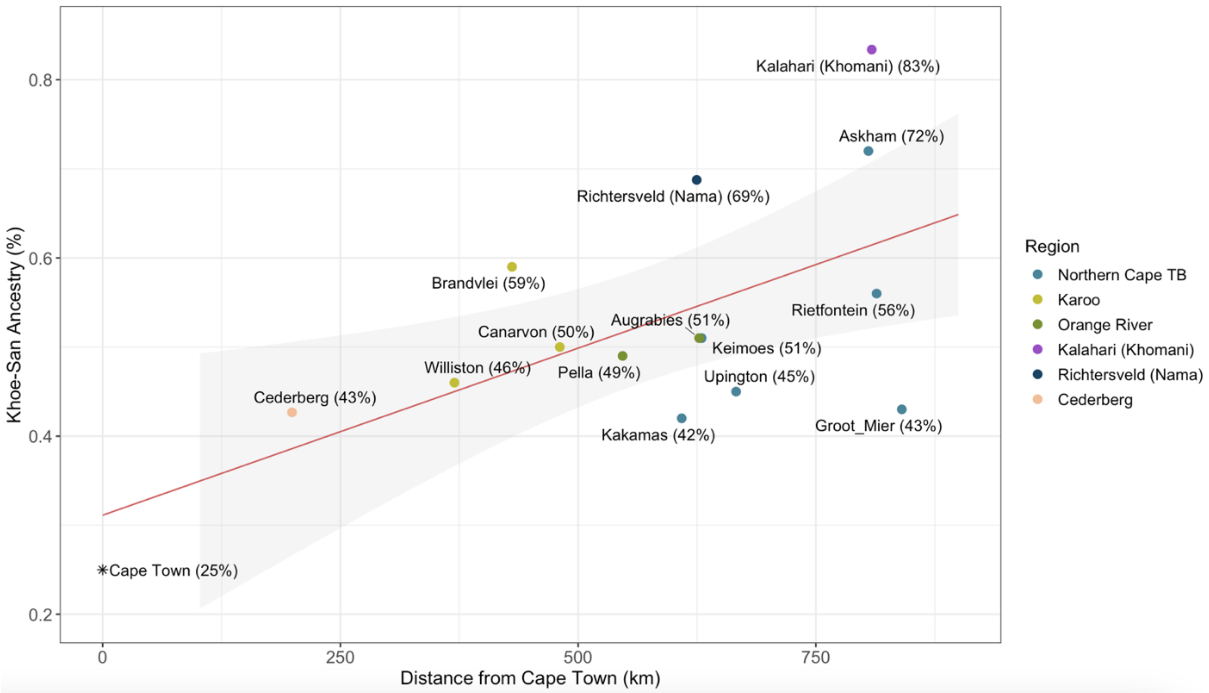

Supplemental Figure 8) Proportion of Khoe-San ancestry by distance from Cape Town. Northern Cape Tuberculosis project participants are separated out by sampling site.

bioRxiv preprint doi: https://doi.org/10.1101/2025.10.05.680541; this version posted October 7, 2025. The copyright holder for this preprint (which was not certified by peer review) is the author/funder, who has granted bioRxiv a license to display the preprint in perpetuity. It is made available under aCC-BY 4.0 International license.

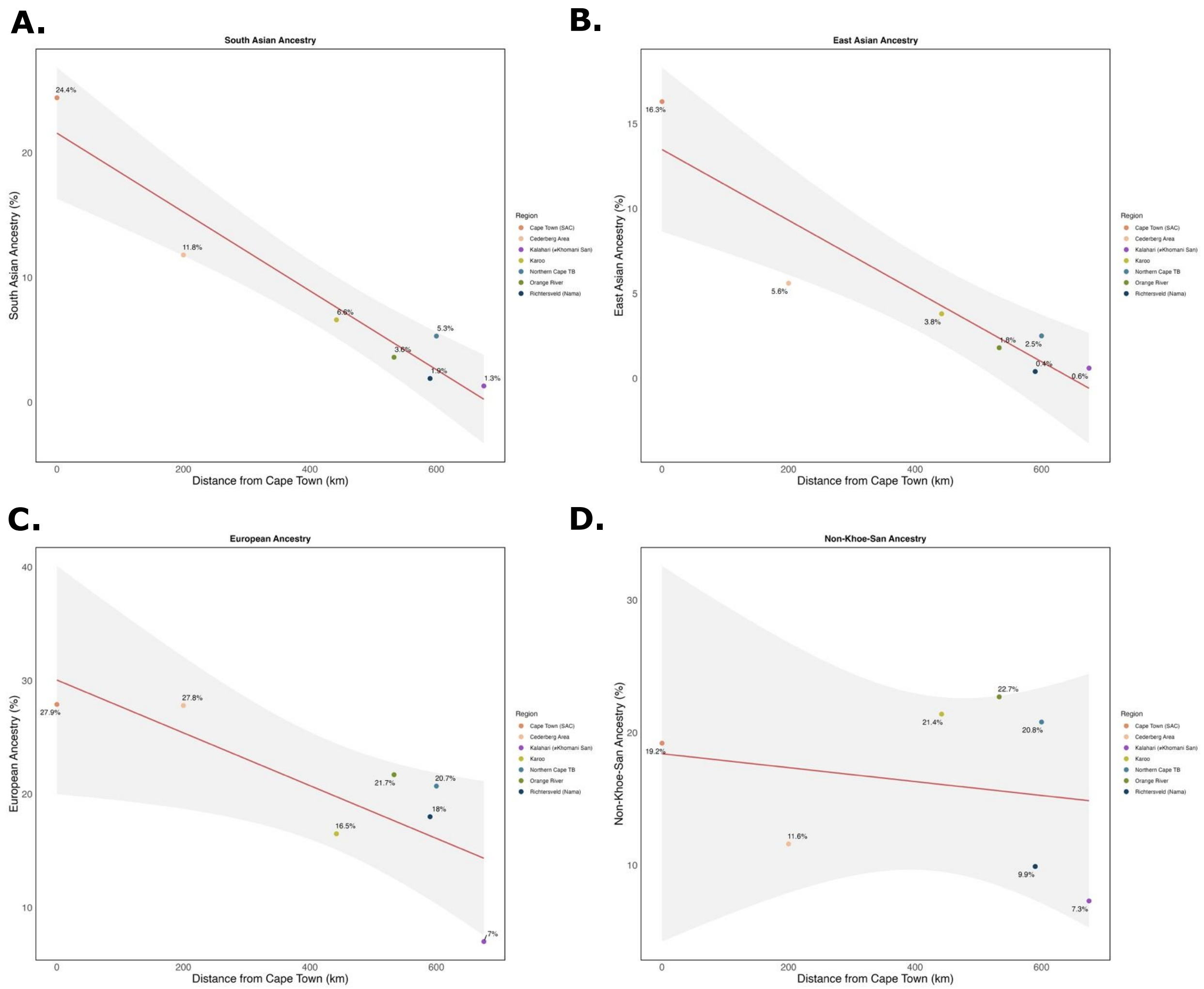

Supplemental Figure 9) Linear regression showing ancestry by distance for A) South Asian (R^2 = 0.895), B) East Asian (R^2 = 0.847), C) European (R^2 = 0.0572), and D) Non-Khoe-San Ancestry (R^2 = 0.004).

bioRxiv preprint doi: https://doi.org/10.1101/2025.10.05.680541; this version posted October 7, 2025. The copyright holder for this preprint (which was not certified by peer review) is the author/funder, who has granted bioRxiv a license to display the preprint in perpetuity. It is made available under aCC-BY 4.0 International license.

Local ancestry results

Our model appears well-calibrated, with the confusion matrix showing >96% accuracy in predicting EAS, NKA, and KHS haplotypes, and ~90% accuracy for EUR and SAS segments. Correlation between ADMIXTURE results for 5 population clusters and Gnomix global ancestry results is high for EUR, NKA, and KHS ($r^2 \geq 0.96$), albeit slightly lower for smaller segments like EAS ($r^2 = 0.94$) and SAS ($r^2 = 0.91$), still indicating a strong correlation.

The highest local ancestry misassignment occurs between SAS and EUR ancestries, with 6.9% of SAS segments being assigned to EUR and approximately 3.5% of EUR segments assigned to SAS. This misassignment also contributes to the larger variance in EUR and SAS ancestry probability distributions (Supplemental Figure 11). We attributed this to the 'Indian cline,' referring to the geographical distribution of two primary ancestral groups across South Asia: the 'Ancestral North Indians' (ANI) and the 'Ancestral South Indians' (ASI)[8–11]. The ANI share closer genetic similarities with populations from the Near Eastern and European populations, while the ASI are more closely related to East Asians[8–11], though many populations across South Asia retain a proportion of both ancestries. The ANI component among the BEB, STU, and GIH is visualized in our ADMIXTURE, highlighted in light green.

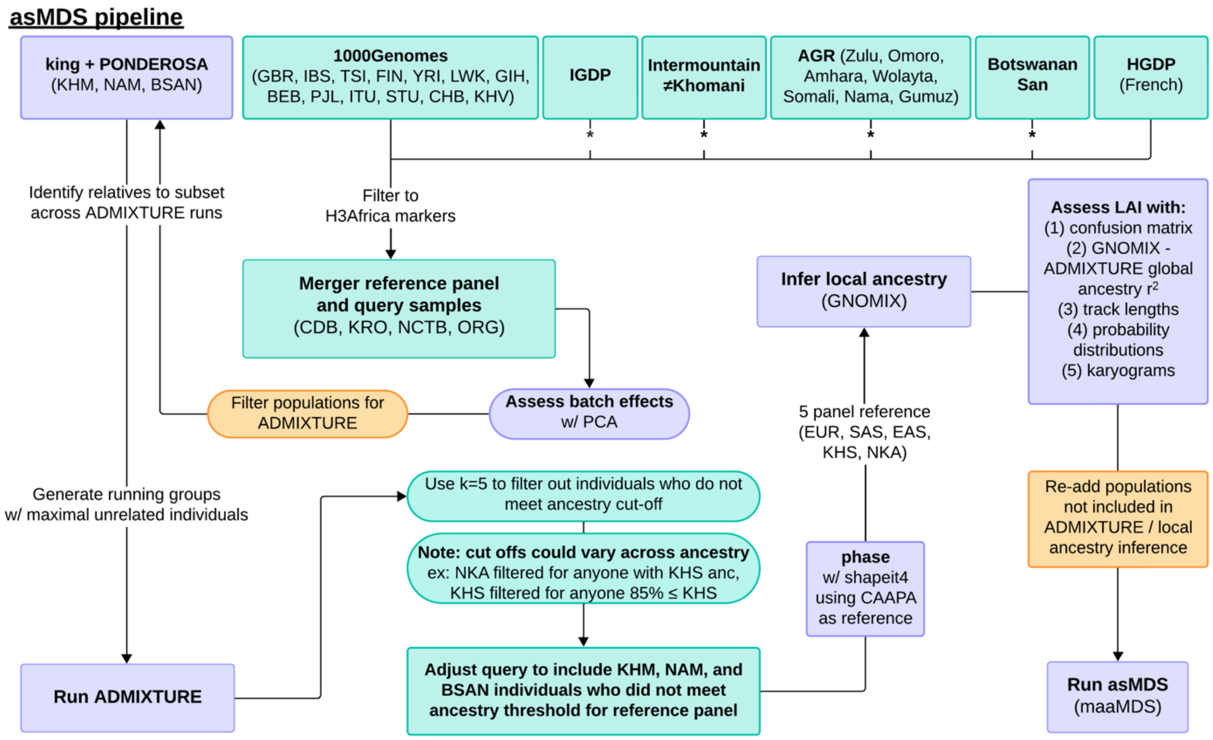

Supplemental Figure 10) Local Ancestry and ancestry-specific MDS pipeline. Shapes in turquoise highlight steps taken regarding sample consolidation and filtering, while orange shapes are steps taken to filter or add entire populations. Shapes in purple major analyses executed throughout the pipeline. The * within lines represent whole genomes that were lifted from HG19 to HG38.

bioRxiv preprint doi: https://doi.org/10.1101/2025.10.05.680541; this version posted October 7, 2025. The copyright holder for this preprint (which was not certified by peer review) is the author/funder, who has granted bioRxiv a license to display the preprint in perpetuity. It is made available under aCC-BY 4.0 International license.

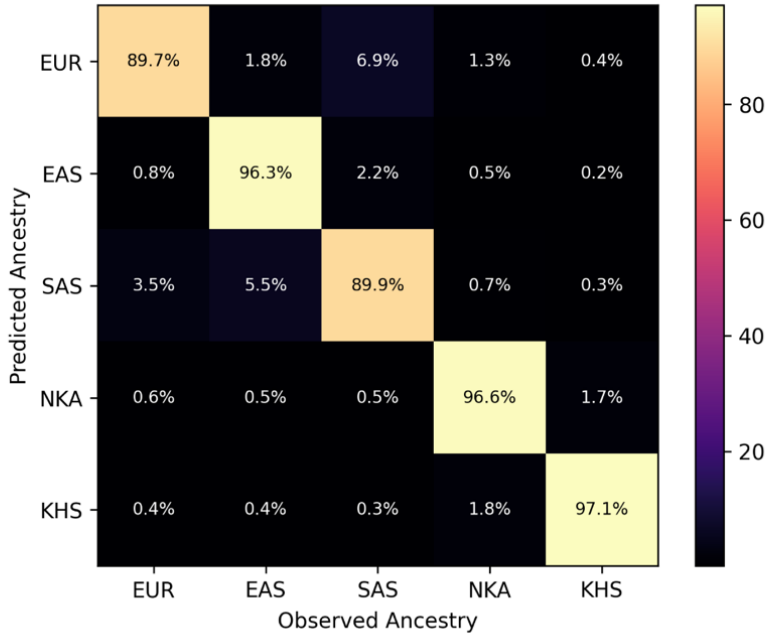

Supplemental Figure 11) Local ancestry inference confusion matrix normalized across all chromosomes, where the x-axis is the ancestry observed, and the y-axis is the model's predicted ancestry. The heat map highlights the accuracy of our model.

bioRxiv preprint doi: https://doi.org/10.1101/2025.10.05.680541; this version posted October 7, 2025. The copyright holder for this preprint (which was not certified by peer review) is the author/funder, who has granted bioRxiv a license to display the preprint in perpetuity. It is made available under aCC-BY 4.0 International license.

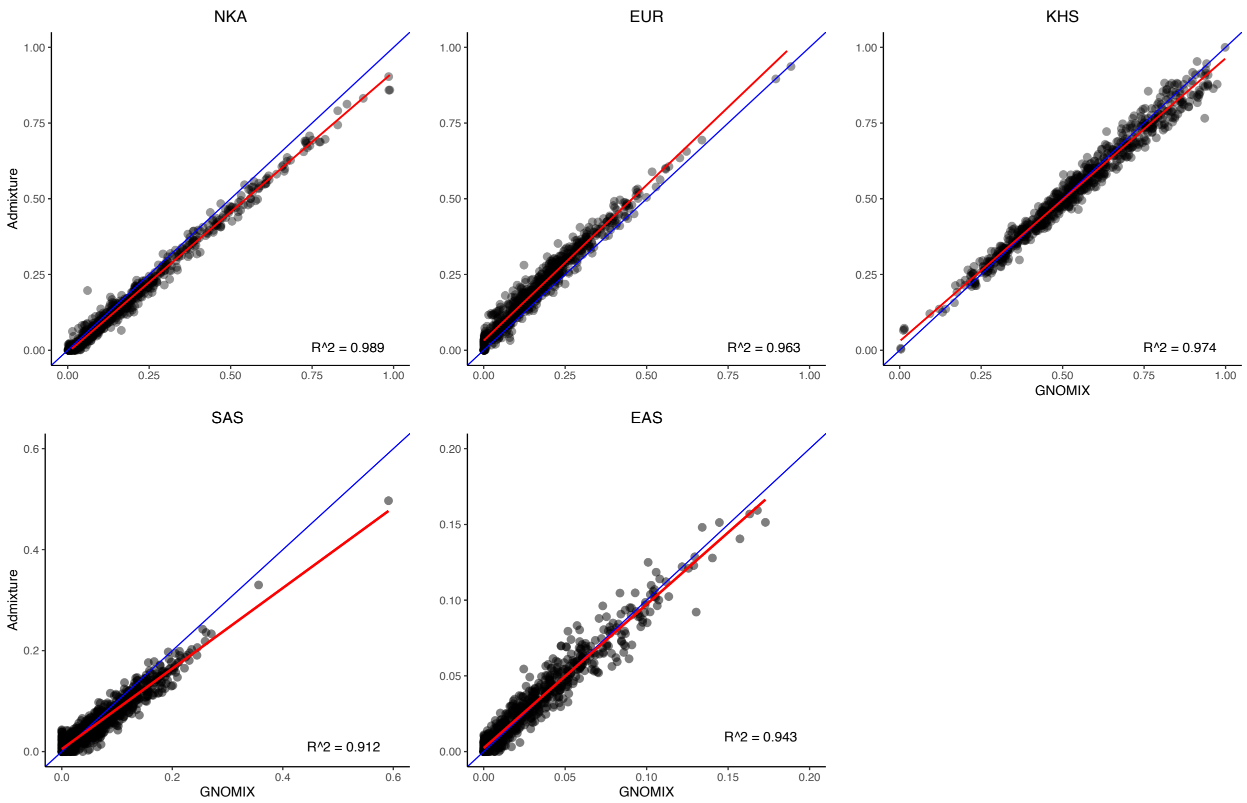

Supplemental Figure 13) Correlation between GNOMIX global ancestry results and ADMIXTURE, where the red line is the best fit and blue line is x=y. R-squared values for each ancestry is reported on the bottom right of the respective plot.

bioRxiv preprint doi: https://doi.org/10.1101/2025.10.05.680541; this version posted October 7, 2025. The copyright holder for this preprint (which was not certified by peer review) is the author/funder, who has granted bioRxiv a license to display the preprint in perpetuity. It is made available under aCC-BY 4.0 International license.

**Pop**	**EAS**	**EUR**	**KHS**	**NKA**	**SAS**
CDB	0.052	0.236	0.447	0.128	0.137
KRO	0.039	0.137	0.517	0.234	0.073
NCTB	0.023	0.174	0.517	0.224	0.062
ORG	0.017	0.176	0.523	0.238	0.045
KHM	0.008	0.067	0.789	0.110	0.026
NAM	0.003	0.116	0.729	0.121	0.032
BotswananSan	0.000	0.013	0.736	0.249	0.002
Zulu	0.000	0.002	0.156	0.841	0.000

Supplemental Figure 12) GNOMIX population-specific global ancestry average proportions.

bioRxiv preprint doi: https://doi.org/10.1101/2025.10.05.680541; this version posted October 7, 2025. The copyright holder for this preprint (which was not certified by peer review) is the author/funder, who has granted bioRxiv a license to display the preprint in perpetuity. It is made available under aCC-BY 4.0 International license.

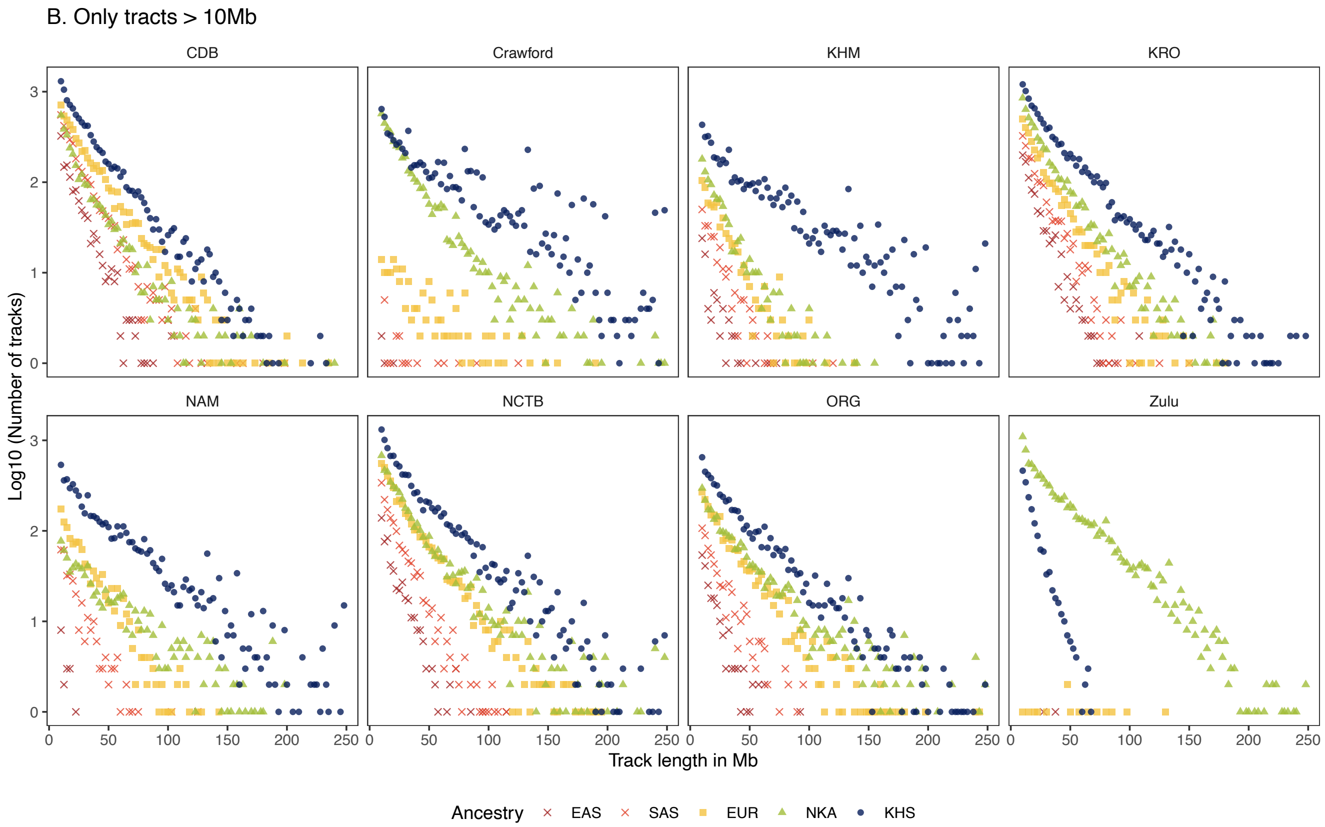

Supplemental Figure 14) Track lengths with at least 75% probability of the respective local ancestry and greater than 10Mb across query populations. The x-axis is the track length in Mb and the y-axis is the log10 of the number of tracks.

bioRxiv preprint doi: https://doi.org/10.1101/2025.10.05.680541; this version posted October 7, 2025. The copyright holder for this preprint (which was not certified by peer review) is the author/funder, who has granted bioRxiv a license to display the preprint in perpetuity. It is made available under aCC-BY 4.0 International license.

Ancestry-specific MDS results

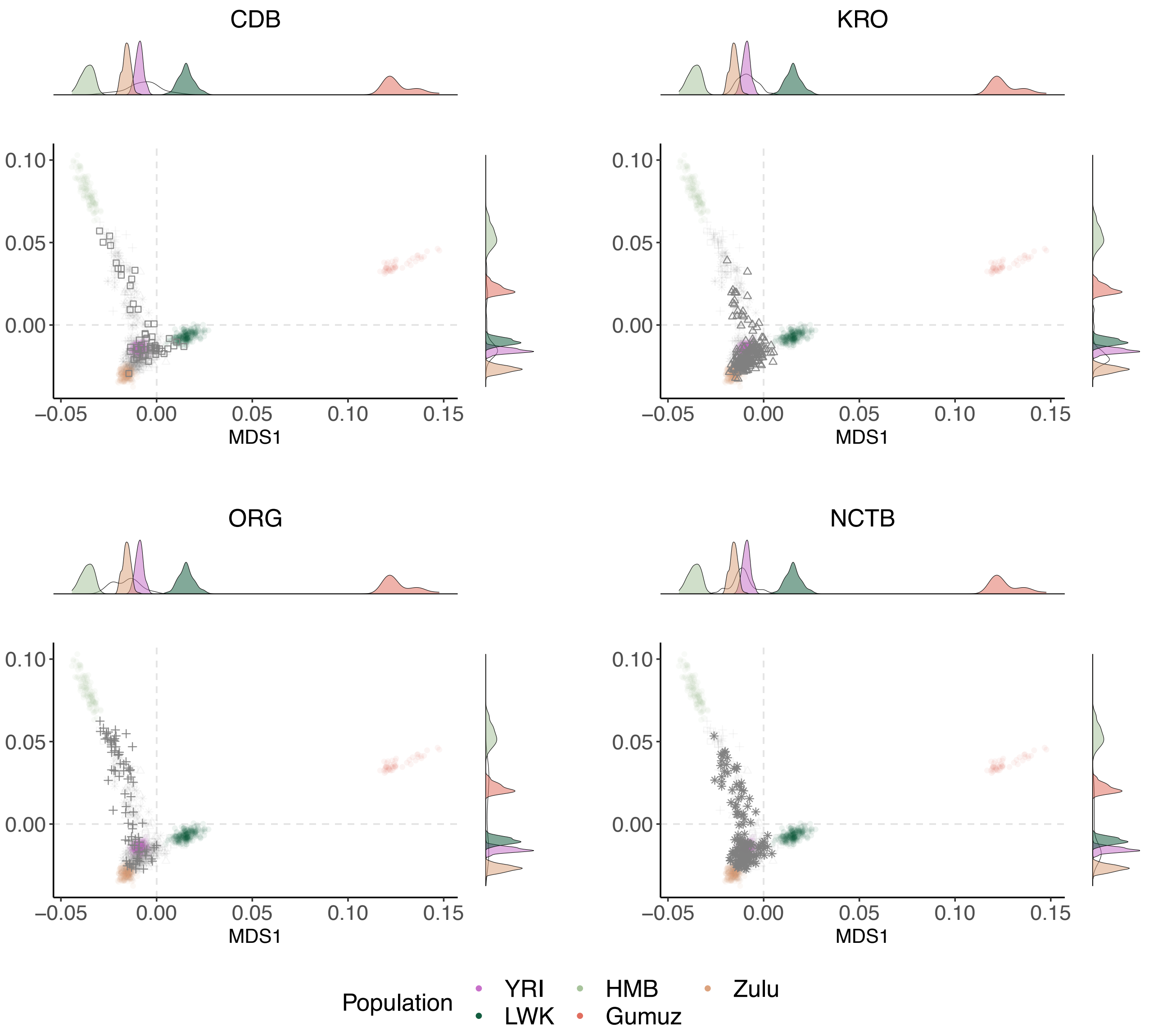

Supplemental Figure 15) NKA asMDS results by population. Density plots for MDS1 and MDS2 are provided with the respective query population density visualized using a transparent line.

bioRxiv preprint doi: https://doi.org/10.1101/2025.10.05.680541; this version posted October 7, 2025. The copyright holder for this preprint (which was not certified by peer review) is the author/funder, who has granted bioRxiv a license to display the preprint in perpetuity. It is made available under aCC-BY 4.0 International license.

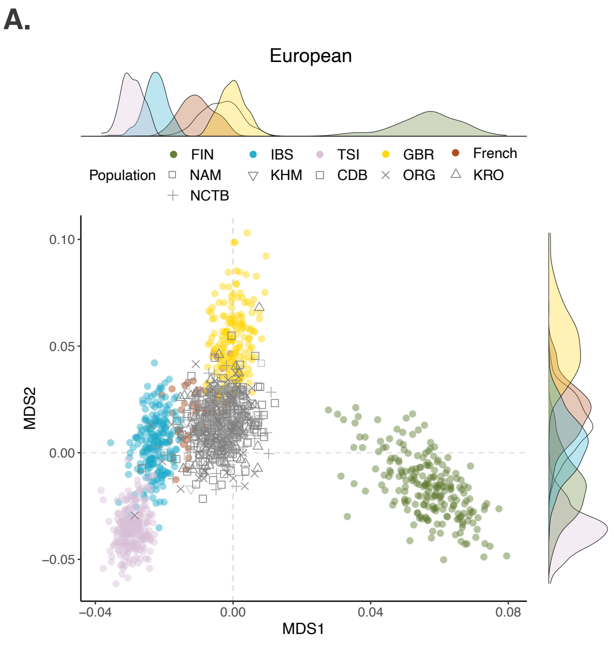

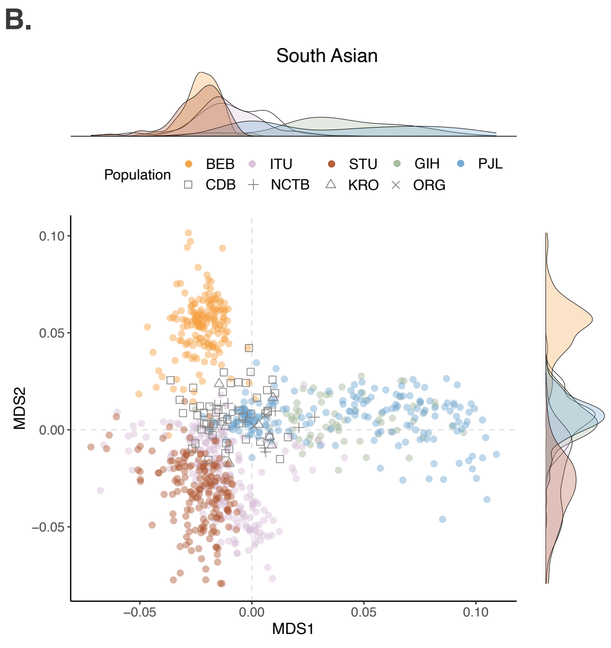

Supplemental Figure 16) European and South Asian asMDS. Local ancestry results were filtered for haplotypes with 75% probability for the respective ancestry and individuals were filtered for at least 20% for the ancestry under investigation.

bioRxiv preprint doi: https://doi.org/10.1101/2025.10.05.680541; this version posted October 7, 2025. The copyright holder for this preprint (which was not certified by peer review) is the author/funder, who has granted bioRxiv a license to display the preprint in perpetuity. It is made available under aCC-BY 4.0 International license.

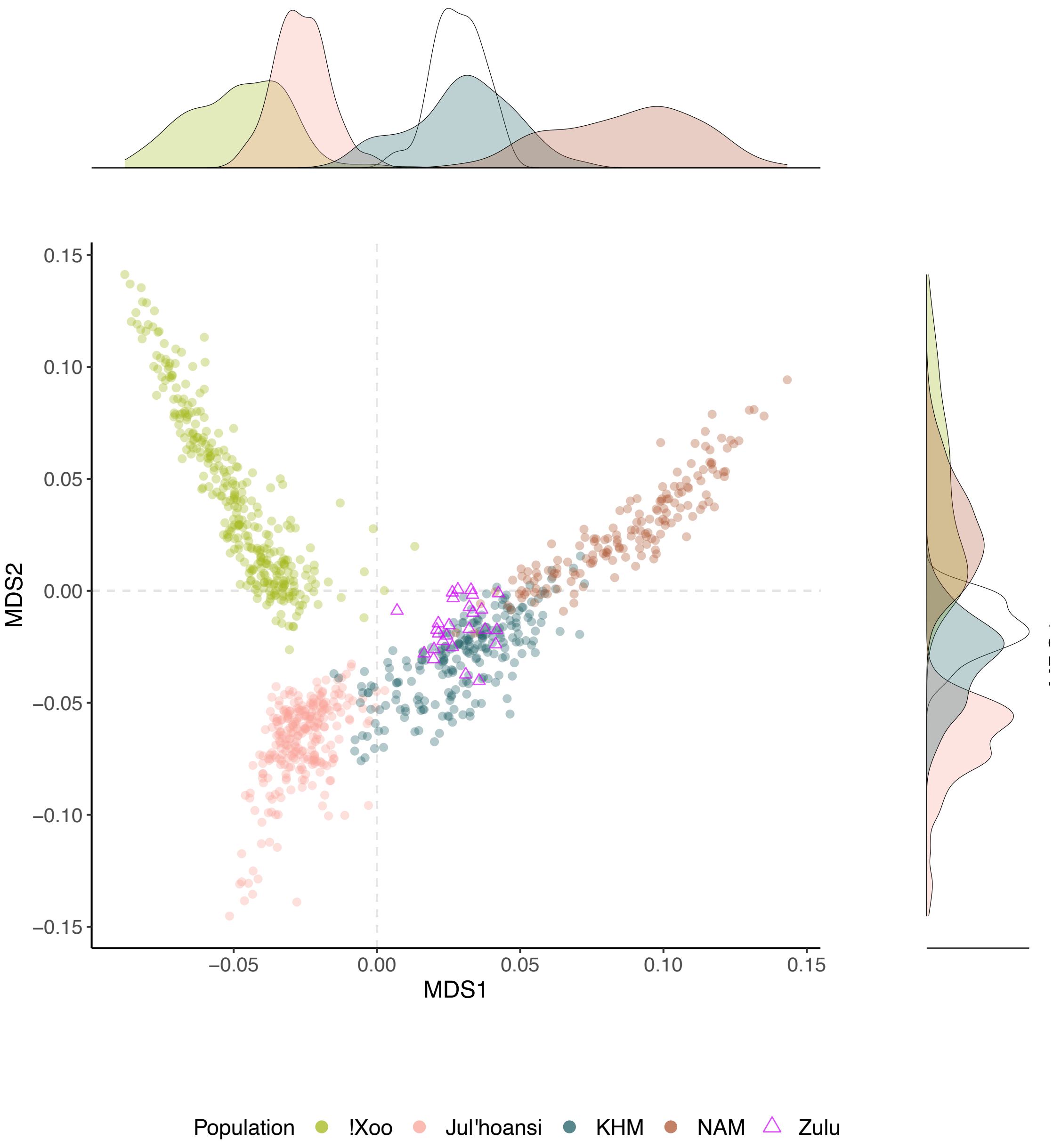

Supplemental Figure 17) Zulu derived Khoe-San asMDS**.**

bioRxiv preprint doi: https://doi.org/10.1101/2025.10.05.680541; this version posted October 7, 2025. The copyright holder for this preprint (which was not certified by peer review) is the author/funder, who has granted bioRxiv a license to display the preprint in perpetuity. It is made available under aCC-BY 4.0 International license.

Admixture Proportions

**Broad_Anc**	**Population**	**n**
Query	Cederberg	170
Query	Karoo	171
Query	Northern Cape TB	183
Query	Middle Orange River	96
East African	Amhara	22
East African	Gumuz	23
East African	Oromo	24
East African	Somali	23
East African	Wolayta	24
East Asian	Han Chinese	103
East Asian	Vietnamese	99
European	British	91
European	Iberian	106
Southeast Asian	Indonesian	130
Khoe-San	Botswanan San	313
Khoe-San	≠Khomani San	115
Khoe-San	Nama	92
Niger-Congo	Himba	123
Niger-Congo	Luhya	99
Niger-Congo	Yoruba	108
Niger-Congo	Zulu	98
South Asian	Bengali	86
South Asian	Gujarati	103
South Asian	Tamil	102

Supplemental Table 1) Sample Size for each population included in ADMIXTURE analysis, as well as their respective broader ancestry (Broad_Anc) category. Newly collected samples have the broader ancestry "Query".

bioRxiv preprint doi: https://doi.org/10.1101/2025.10.05.680541; this version posted October 7, 2025. The copyright holder for this preprint (which was not certified by peer review) is the author/funder, who has granted bioRxiv a license to display the preprint in perpetuity. It is made available under aCC-BY 4.0 International license.

Pop	SAS	EAS	NKA	KHS	EUR
CDB	0.118	0.056	0.116	0.432	0.278
KRO	0.066	0.038	0.214	0.518	0.165
ORG	0.036	0.018	0.227	0.502	0.217
NCTB	0.053	0.025	0.208	0.507	0.207
KHM	0.013	0.006	0.073	0.838	0.070
NAM	0.019	0.004	0.099	0.699	0.180
Crawford	0.002	0.001	0.129	0.859	0.009
Amhara	0.033	0.014	0.395	0.040	0.517
BEB	0.753	0.136	0.000	0.000	0.111
CHB	0.001	0.992	0.000	0.000	0.007
GBR	0.002	0.000	0.000	0.000	0.998
GIH	0.818	0.004	0.000	0.000	0.178
Gumuz	0.032	0.036	0.742	0.084	0.106
HMB	0.005	0.006	0.951	0.031	0.007
IBS	0.002	0.000	0.014	0.000	0.983
IND	0.116	0.863	0.011	0.009	0.001
KHV	0.012	0.986	0.000	0.000	0.002
LWK	0.015	0.011	0.887	0.036	0.050
Oromo	0.029	0.017	0.420	0.051	0.483
STU	0.864	0.040	0.000	0.000	0.096
Somali	0.013	0.018	0.507	0.036	0.425
Wolayta	0.035	0.020	0.460	0.081	0.404
YRI	0.015	0.008	0.956	0.005	0.016
Zulu	0.007	0.004	0.794	0.182	0.012

Supplemental Table 2) ADMIXTURE k=5

bioRxiv preprint doi: https://doi.org/10.1101/2025.10.05.680541; this version posted October 7, 2025. The copyright holder for this preprint (which was not certified by peer review) is the author/funder, who has granted bioRxiv a license to display the preprint in perpetuity. It is made available under aCC-BY 4.0 International license.

Pop	EAFR	EUR	SAS	IND	WBNT	EBNT	KHS	EAS
CDB	0.024	0.266	0.117	0.031	0.015	0.097	0.420	0.031
KRO	0.010	0.158	0.063	0.021	0.010	0.212	0.505	0.021
ORG	0.034	0.207	0.034	0.010	0.095	0.124	0.486	0.010
NCTB	0.020	0.199	0.051	0.013	0.034	0.175	0.494	0.014
KHM	0.015	0.066	0.013	0.003	0.006	0.075	0.819	0.004
NAM	0.065	0.160	0.017	0.002	0.056	0.031	0.668	0.001
Crawford	0.015	0.007	0.001	0.000	0.039	0.100	0.838	0.000
Amhara	0.574	0.413	0.010	0.001	0.000	0.000	0.002	0.000
BEB	0.000	0.064	0.793	0.030	0.000	0.000	0.000	0.112
CHB	0.000	0.005	0.004	0.002	0.000	0.000	0.000	0.989
GBR	0.000	0.997	0.003	0.000	0.000	0.000	0.000	0.000
GIH	0.000	0.126	0.868	0.002	0.000	0.000	0.000	0.003
Gumuz	0.833	0.000	0.000	0.000	0.007	0.127	0.032	0.001
HMB	0.012	0.003	0.003	0.002	0.872	0.092	0.014	0.002
IBS	0.023	0.974	0.002	0.000	0.000	0.001	0.000	0.000
IND	0.001	0.001	0.011	0.617	0.000	0.000	0.000	0.369
KHV	0.000	0.001	0.006	0.086	0.000	0.000	0.000	0.907
LWK	0.248	0.008	0.002	0.002	0.075	0.640	0.022	0.002
Oromo	0.603	0.376	0.008	0.001	0.000	0.002	0.010	0.000
STU	0.001	0.044	0.908	0.029	0.000	0.000	0.000	0.018
Somali	0.693	0.292	0.001	0.000	0.001	0.012	0.000	0.000
Wolayta	0.647	0.293	0.012	0.002	0.002	0.005	0.038	0.001
YRI	0.108	0.001	0.002	0.002	0.121	0.764	0.001	0.002
Zulu	0.011	0.007	0.002	0.001	0.010	0.787	0.181	0.002

Supplemental Table 3) ADMIXTURE k=8

bioRxiv preprint doi: https://doi.org/10.1101/2025.10.05.680541; this version posted October 7, 2025. The copyright holder for this preprint (which was not certified by peer review) is the author/funder, who has granted bioRxiv a license to display the preprint in perpetuity. It is made available under aCC-BY 4.0 International license.

**Pop**	**S_SAN**	**WBNT**	**EAS**	**EAFR**	**IND1**	**SAS**	**N_SAN**	**EUR**	**EBNT**	**IND2**
CDB	0.420	0.018	0.014	0.020	0.030	0.121	0.026	0.241	0.093	0.017
KRO	0.466	0.012	0.008	0.008	0.023	0.065	0.068	0.133	0.207	0.011
ORG	0.468	0.098	0.007	0.029	0.008	0.036	0.047	0.180	0.120	0.006
NCTB	0.431	0.036	0.006	0.017	0.014	0.053	0.090	0.177	0.169	0.007
KHM	0.600	0.006	0.002	0.011	0.003	0.013	0.255	0.042	0.066	0.002
NAM	0.696	0.057	0.002	0.054	0.001	0.019	0.022	0.119	0.030	0.001
Crawford	0.100	0.016	0.000	0.013	0.000	0.001	0.764	0.007	0.099	0.000
Amhara	0.003	0.000	0.000	0.575	0.000	0.010	0.000	0.410	0.000	0.000
BEB	0.000	0.000	0.090	0.000	0.034	0.793	0.000	0.064	0.000	0.019
CHB	0.000	0.000	0.951	0.000	0.045	0.001	0.000	0.002	0.000	0.000
GBR	0.000	0.000	0.000	0.000	0.000	0.003	0.000	0.997	0.000	0.000
GIH	0.000	0.000	0.002	0.000	0.002	0.868	0.000	0.127	0.000	0.002
Gumuz	0.000	0.003	0.000	0.830	0.000	0.000	0.038	0.000	0.128	0.000
HMB	0.009	0.871	0.002	0.012	0.002	0.002	0.007	0.002	0.090	0.002
IBS	0.000	0.000	0.000	0.023	0.000	0.001	0.000	0.974	0.001	0.000
IND	0.000	0.000	0.077	0.000	0.459	0.012	0.000	0.001	0.000	0.450
KHV	0.000	0.000	0.651	0.000	0.335	0.008	0.000	0.001	0.000	0.005
LWK	0.008	0.068	0.001	0.253	0.002	0.002	0.016	0.011	0.638	0.002
Oromo	0.009	0.000	0.000	0.603	0.001	0.008	0.004	0.373	0.002	0.001
STU	0.000	0.000	0.013	0.001	0.013	0.908	0.000	0.044	0.000	0.022
Somali	0.001	0.001	0.000	0.696	0.000	0.001	0.000	0.289	0.012	0.000
Wolayta	0.021	0.001	0.001	0.647	0.001	0.012	0.021	0.289	0.005	0.002
YRI	0.001	0.112	0.002	0.114	0.001	0.003	0.001	0.002	0.761	0.003
Zulu	0.167	0.008	0.001	0.013	0.001	0.001	0.023	0.004	0.780	0.001

Supplemental Table 4) ADMIXTURE k=10

bioRxiv preprint doi: https://doi.org/10.1101/2025.10.05.680541; this version posted October 7, 2025. The copyright holder for this preprint (which was not certified by peer review) is the author/funder, who has granted bioRxiv a license to display the preprint in perpetuity. It is made available under aCC-BY 4.0 International license.

Population Centroids and Euclidean Distances

We aim to best characterize specific source populations for the five primary ancestries within the admixed study populations. We further investigated the relationships among populations by computing the Euclidean distances between each population's centroid (i.e., a population's mean coordinates along MDS1 and MDS2; see *Supplemental Methods*). For instance, the distances from the ≠Khomani San to the Cederberg (CDB), Karoo (KRO), and Orange River (ORG) populations are 0.036, 0.022, and 0.034, respectively, whereas the corresponding distances from NAM to these populations are 0.043, 0.059, and 0.046. These results suggest that, although Cederberg, Karoo, and Orange River are relatively similar in their overall MDS distribution, they share a more similar Khoe-San ancestry profile with ≠Khomani San than with the Nama. The Karoo (0.022) and NCTB (0.021) show the smallest distance to ≠Khomani San, indicating particularly close clustering. Additionally, Zulu haplotypes fall with the ≠Khomani San (0.010), suggesting that the Khoe-San haplotypes present in Zulu individuals are genetically similar to ≠Khomani San.

Among the query populations, we observe generally small distances to Yoruba (YRI), Luhya (LWK), and Zulu, compared to the Himba (HMB). Haplotypes from individuals sampled in the Karoo (0.002) demonstrate close similarities to Yoruba, while the Cederberg centroid appears slightly further from the Yoruba (0.009) and Luhya (0.017) (Supplemental Table 2). Participants sampled along Middle Orange River are more distant from the Yoruba (0.028) and Zulu (0.043), but still more similar to these groups than to Himba (0.072). Overall, these patterns indicate that the Cederberg, Karoo, and Orange River populations share closer ancestry profiles with Yoruba, Luhya, and Zulu than with Himba. Similarly, ≠Khomani San NKA haplotype centroid is closest to the Yoruba (0.004) and moderately more distant to the Zulu (0.021) and Luhya (0.025), with increasing distance to Himba (0.095). In contrast, the Nama NKA haplotype centroid is moderately distant to all NKA reference groups: Yoruba (0.036), Zulu (0.051), Luhya (0.040), Himba (0.064).

bioRxiv preprint doi: https://doi.org/10.1101/2025.10.05.680541; this version posted October 7, 2025. The copyright holder for this preprint (which was not certified by peer review) is the author/funder, who has granted bioRxiv a license to display the preprint in perpetuity. It is made available under aCC-BY 4.0 International license.

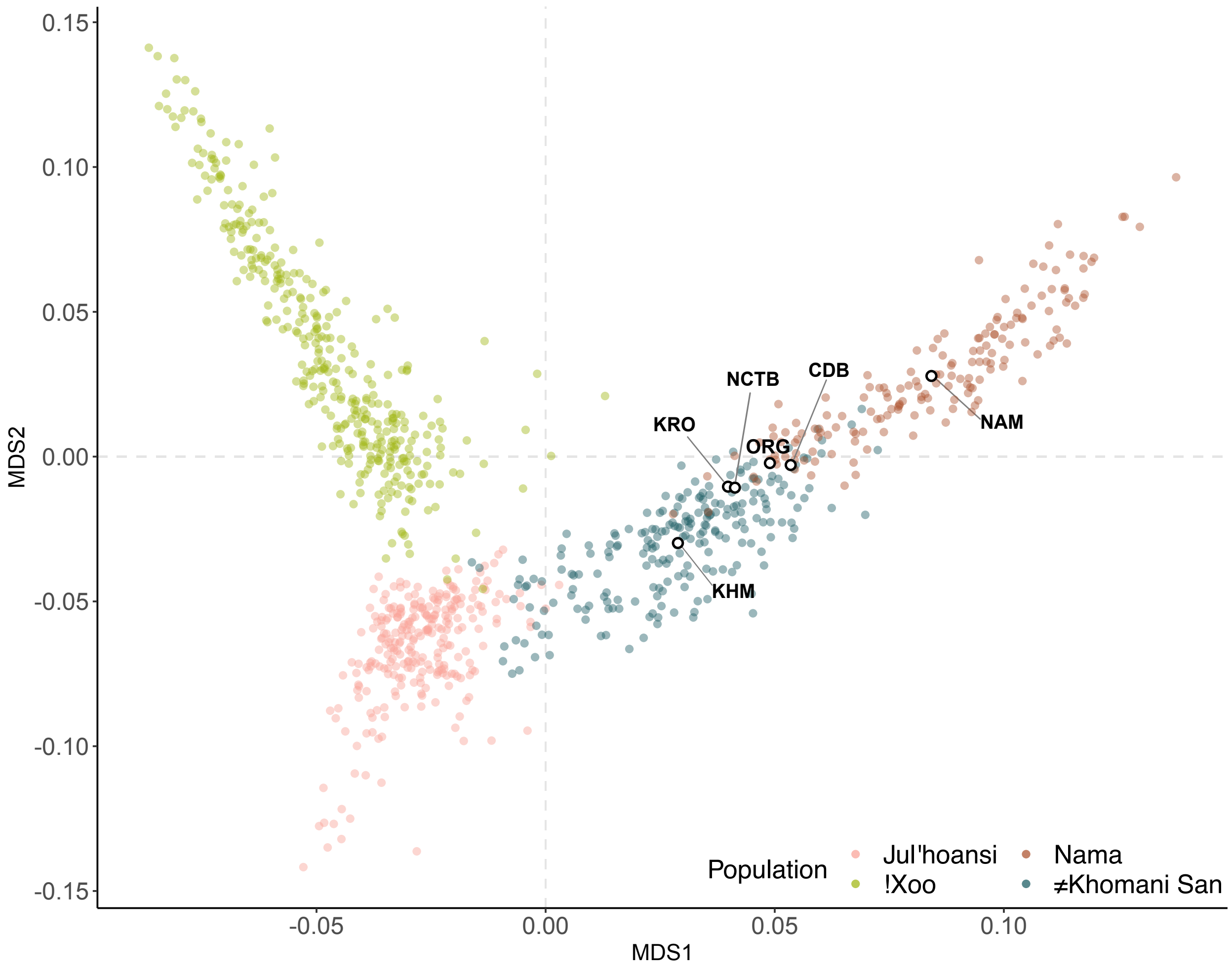

Supplemental Figure 18) Khoe-San derived population centroids plotted against reference populations. Query sample haplotypes are not plotted for clearer visualization of reference populations distributions.

bioRxiv preprint doi: https://doi.org/10.1101/2025.10.05.680541; this version posted October 7, 2025. The copyright holder for this preprint (which was not certified by peer review) is the author/funder, who has granted bioRxiv a license to display the preprint in perpetuity. It is made available under aCC-BY 4.0 International license.

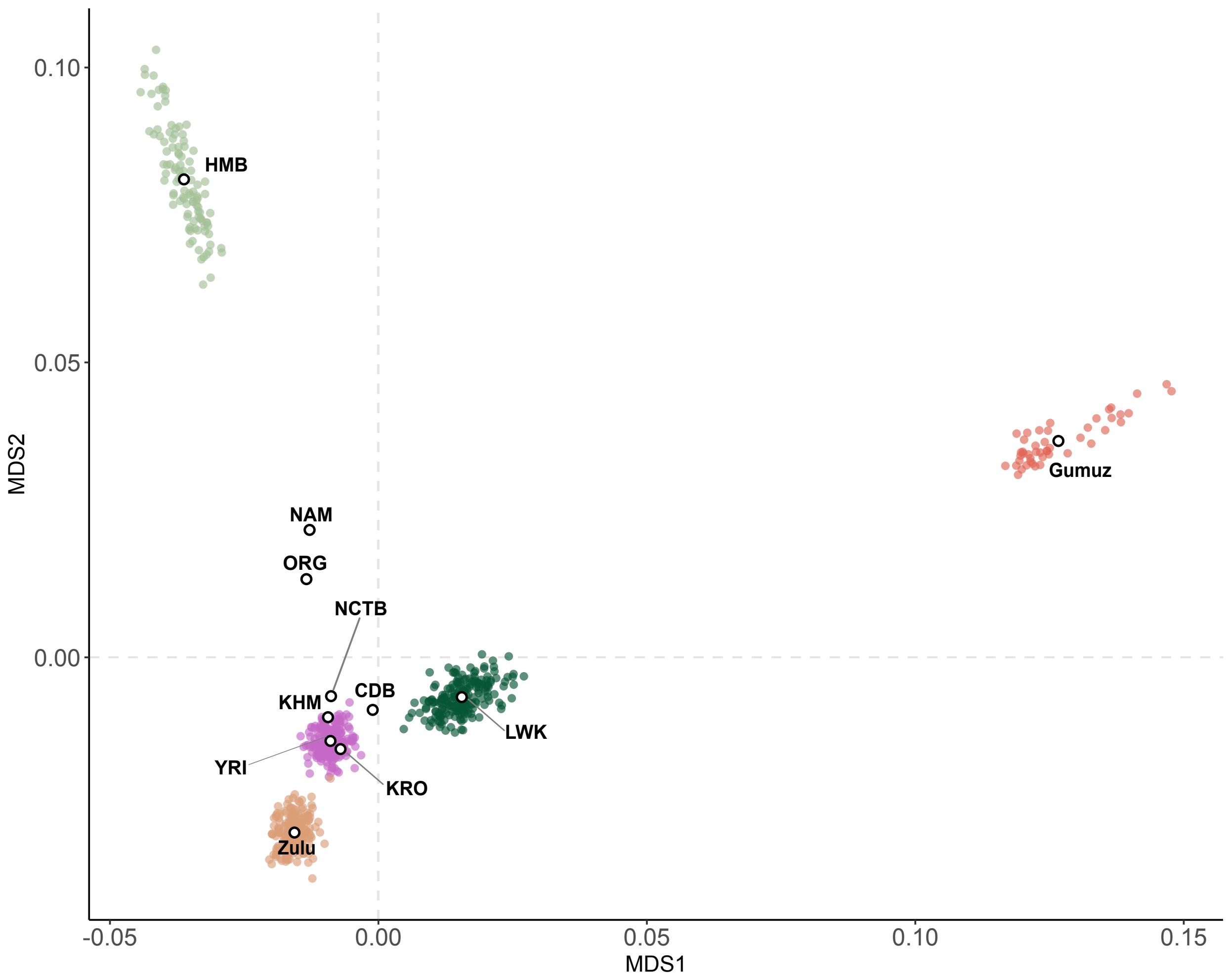

Supplemental Figure 19) Non-Khoe-San derived population centroids plotted against non-Khoe-San reference populations. Query sample haplotypes are not plotted for clearer visualization of reference populations distributions.

bioRxiv preprint doi: https://doi.org/10.1101/2025.10.05.680541; this version posted October 7, 2025. The copyright holder for this preprint (which was not certified by peer review) is the author/funder, who has granted bioRxiv a license to display the preprint in perpetuity. It is made available under aCC-BY 4.0 International license.

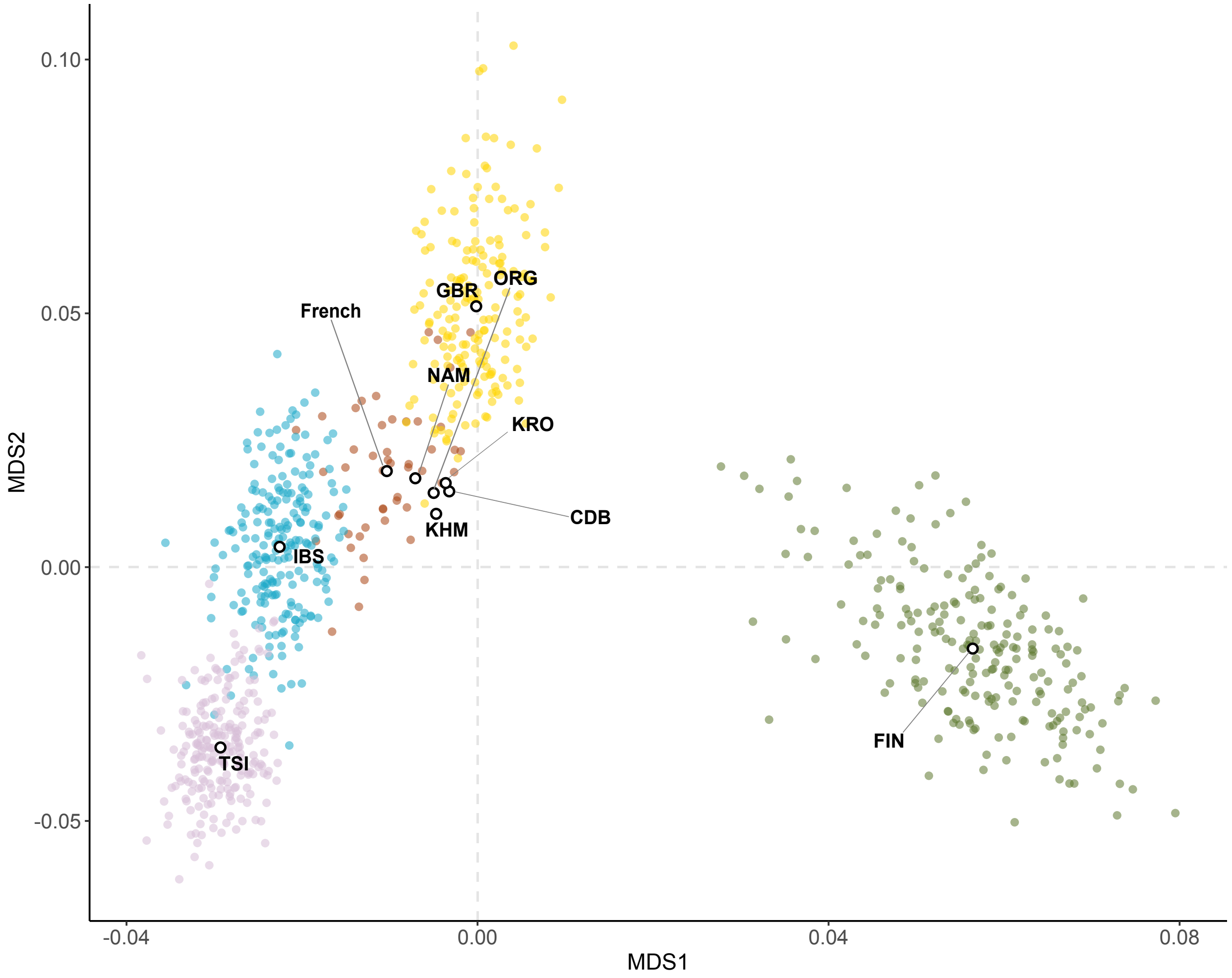

Supplemental Figure 20) European derived centroids plotted against reference populations. Query sample haplotypes are not plotted for clearer visualization of reference populations distributions.

bioRxiv preprint doi: https://doi.org/10.1101/2025.10.05.680541; this version posted October 7, 2025. The copyright holder for this preprint (which was not certified by peer review) is the author/funder, who has granted bioRxiv a license to display the preprint in perpetuity. It is made available under aCC-BY 4.0 International license.

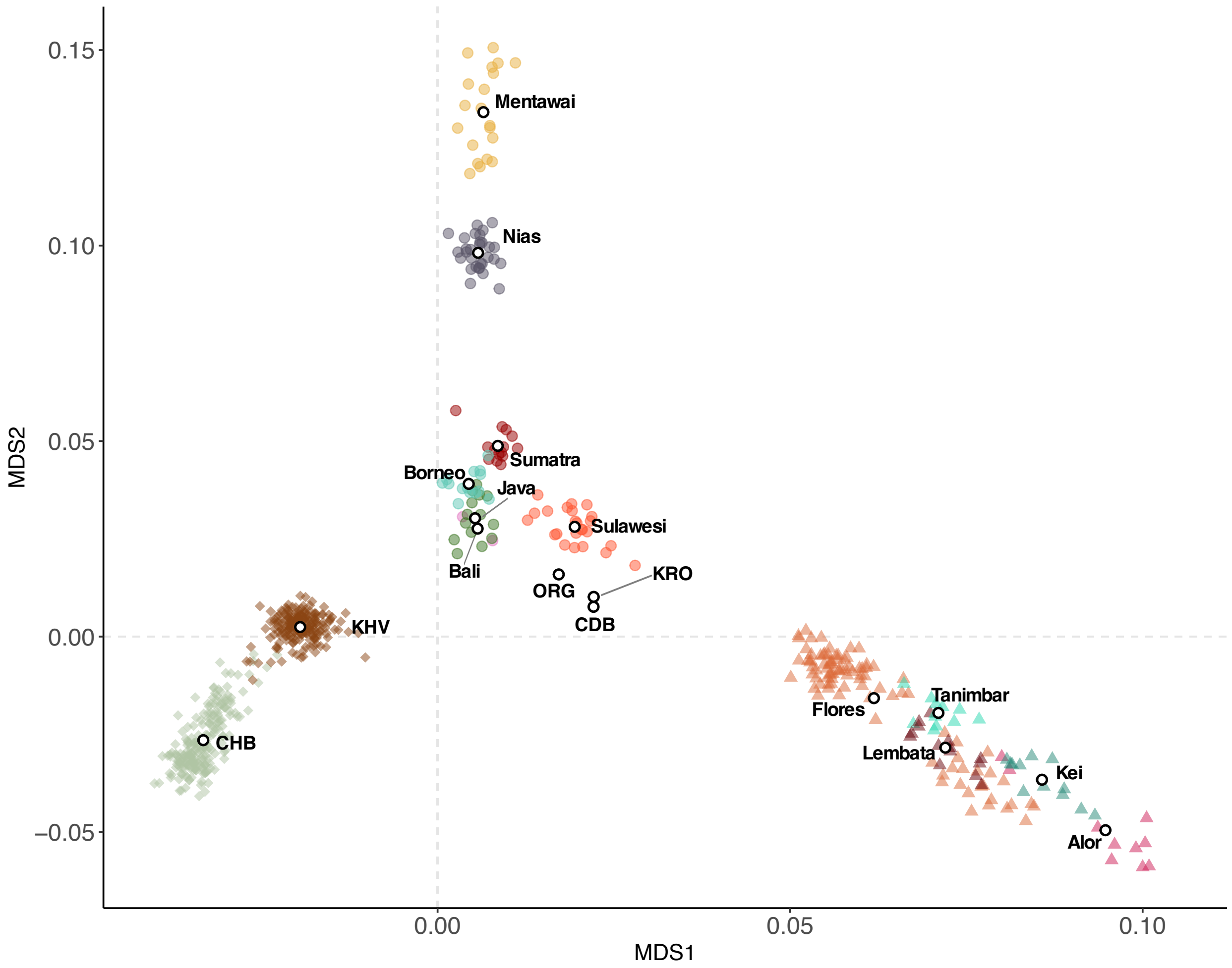

Supplemental Figure 21) Other Asian derived centroids plotted against reference populations. Query sample haplotypes are not plotted for clearer visualization of reference populations distributions.

bioRxiv preprint doi: https://doi.org/10.1101/2025.10.05.680541; this version posted October 7, 2025. The copyright holder for this preprint (which was not certified by peer review) is the author/funder, who has granted bioRxiv a license to display the preprint in perpetuity. It is made available under aCC-BY 4.0 International license.

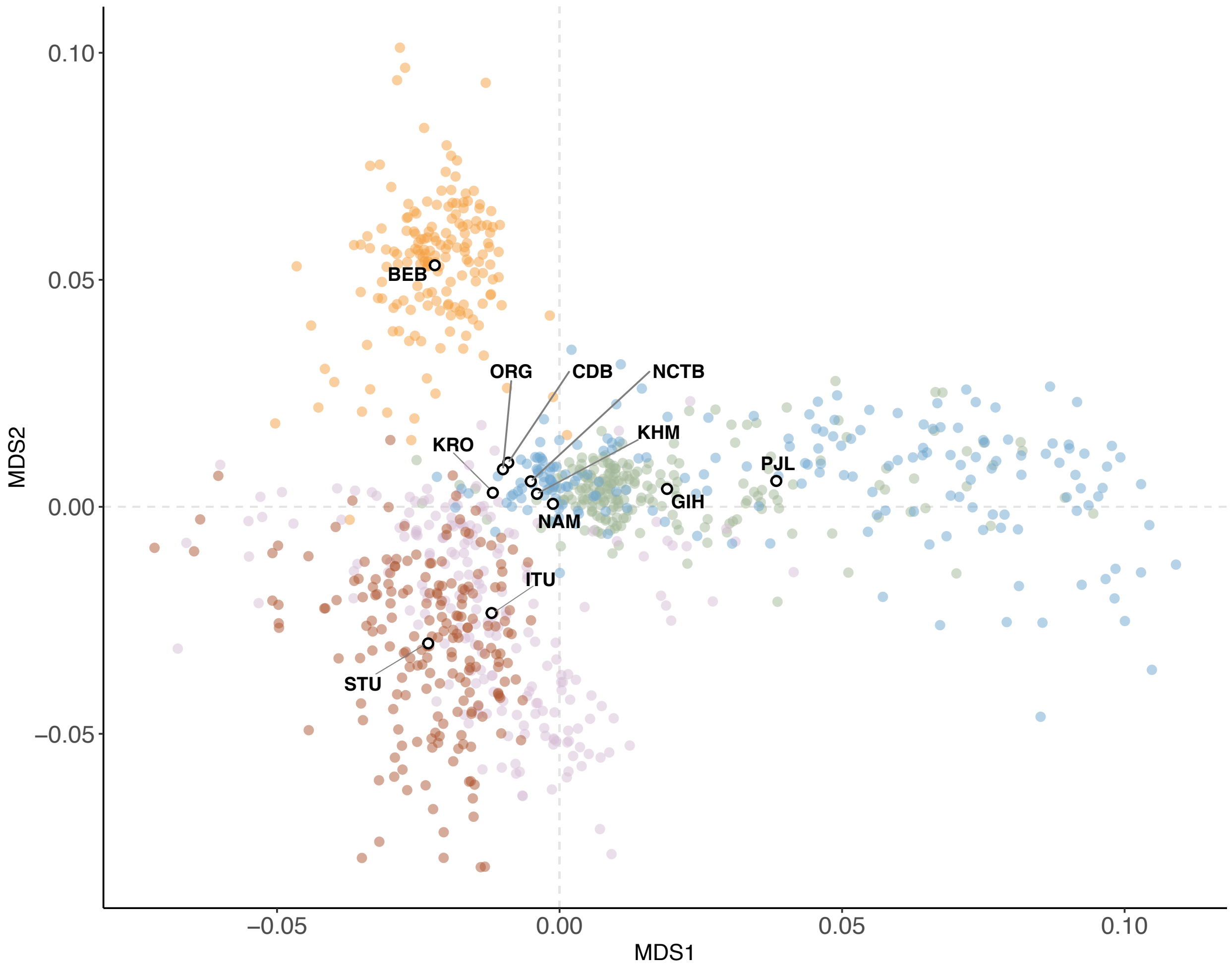

Supplemental Figure 22) South Asian derived centroids plotted against reference populations. Query sample haplotypes are not plotted for clearer visualization of reference populations distributions.

bioRxiv preprint doi: https://doi.org/10.1101/2025.10.05.680541; this version posted October 7, 2025. The copyright holder for this preprint (which was not certified by peer review) is the author/funder, who has granted bioRxiv a license to display the preprint in perpetuity. It is made available under aCC-BY 4.0 International license.

	KHM	NAM	CDB	KRO	ORG	NCTB	!Xoo	Jul'hoansi	Zulu
KHM	0.000	0.080	0.036	0.022	0.034	0.023	0.100	0.067	0.010
NAM	0.080	0.000	0.043	0.059	0.046	0.058	0.131	0.146	0.073
CDB	0.036	0.043	0.000	0.016	0.005	0.014	0.108	0.103	0.030
KRO	0.022	0.059	0.016	0.000	0.012	0.002	0.098	0.087	0.014
ORG	0.034	0.046	0.005	0.012	0.000	0.011	0.103	0.099	0.027
NCTB	0.023	0.058	0.014	0.002	0.011	0.000	0.100	0.088	0.015
!Xoo	0.100	0.131	0.108	0.098	0.103	0.100	0.000	0.104	0.094
Jul'hoansi	0.067	0.146	0.103	0.087	0.099	0.088	0.104	0.000	0.073
Zulu	0.010	0.073	0.030	0.014	0.027	0.015	0.094	0.073	0.000

Supplemental Table 5) Khoe-San Euclidean distances between population centroids using Khoe-San ancestry-specific MDS1 and MDS2 coordinates.

	KHM	NAM	CDB	KRO	ORG	NCTB	Gumuz	HMB	LWK	YRI	Zulu
KHM	0.000	0.032	0.008	0.006	0.024	0.004	0.144	0.095	0.025	0.004	0.021
NAM	0.032	0.000	0.033	0.038	0.008	0.028	0.140	0.064	0.040	0.036	0.051
CDB	0.008	0.033	0.000	0.009	0.025	0.008	0.136	0.097	0.017	0.009	0.025
KRO	0.006	0.038	0.009	0.000	0.030	0.009	0.144	0.101	0.024	0.002	0.017
ORG	0.024	0.008	0.025	0.030	0.000	0.020	0.142	0.072	0.035	0.028	0.043
NCTB	0.004	0.028	0.008	0.009	0.020	0.000	0.142	0.092	0.024	0.008	0.024
Gumuz	0.144	0.140	0.136	0.144	0.142	0.142	0.000	0.169	0.119	0.145	0.157
HMB	0.095	0.064	0.097	0.101	0.072	0.092	0.169	0.000	0.102	0.099	0.113
LWK	0.025	0.040	0.017	0.024	0.035	0.024	0.119	0.102	0.000	0.026	0.039
YRI	0.004	0.036	0.009	0.002	0.028	0.008	0.145	0.099	0.026	0.000	0.017
Zulu	0.021	0.051	0.025	0.017	0.043	0.024	0.157	0.113	0.039	0.017	0.000

Supplemental Table 6) Non-Khoe-San African Euclidean distances between population centroids using non-Khoe-San African ancestry-specific MDS1 and MDS2 coordinates.

bioRxiv preprint doi: https://doi.org/10.1101/2025.10.05.680541; this version posted October 7, 2025. The copyright holder for this preprint (which was not certified by peer review) is the author/funder, who has granted bioRxiv a license to display the preprint in perpetuity. It is made available under aCC-BY 4.0 International license.

	KHM	NAM	CDB	KRO	ORG	NCTB	FIN	French	GBR	IBS	TSI
KHM	0.000	0.005	0.003	0.004	0.002	0.003	0.066	0.011	0.042	0.020	0.052
NAM	0.005	0.000	0.003	0.003	0.003	0.003	0.070	0.006	0.037	0.020	0.055
CDB	0.003	0.003	0.000	0.001	0.002	0.000	0.067	0.009	0.038	0.021	0.055
KRO	0.004	0.003	0.001	0.000	0.002	0.001	0.067	0.008	0.037	0.022	0.056
ORG	0.002	0.003	0.002	0.002	0.000	0.001	0.067	0.009	0.040	0.020	0.054
NCTB	0.003	0.003	0.000	0.001	0.001	0.000	0.067	0.009	0.039	0.021	0.055
FIN	0.066	0.070	0.067	0.067	0.067	0.067	0.000	0.075	0.088	0.082	0.088
French	0.011	0.006	0.009	0.008	0.009	0.009	0.075	0.000	0.034	0.019	0.058
GBR	0.042	0.037	0.038	0.037	0.040	0.039	0.088	0.034	0.000	0.052	0.092
IBS	0.020	0.020	0.021	0.022	0.020	0.021	0.082	0.019	0.052	0.000	0.040
TSI	0.052	0.055	0.055	0.056	0.054	0.055	0.088	0.058	0.092	0.040	0.000

Supplemental Table 7) European Euclidean distances between population centroids using European ancestry-specific MDS1 and MDS2 coordinates.

	KHM	NAM	CDB	KRO	ORG	NCTB	BEB	GIH	ITU	PJL	STU
KHM	0.000	0.004	0.009	0.008	0.008	0.003	0.054	0.023	0.027	0.042	0.038
NAM	0.004	0.000	0.012	0.011	0.012	0.006	0.057	0.020	0.026	0.040	0.038
CDB	0.009	0.012	0.000	0.007	0.002	0.006	0.045	0.029	0.033	0.048	0.042
KRO	0.008	0.011	0.007	0.000	0.005	0.007	0.051	0.031	0.026	0.050	0.035
ORG	0.008	0.012	0.002	0.005	0.000	0.006	0.047	0.029	0.032	0.048	0.041
NCTB	0.003	0.006	0.006	0.007	0.006	0.000	0.051	0.024	0.030	0.043	0.040
BEB	0.054	0.057	0.045	0.051	0.047	0.051	0.000	0.064	0.077	0.077	0.083
GIH	0.023	0.020	0.029	0.031	0.029	0.024	0.064	0.000	0.041	0.019	0.054
ITU	0.027	0.026	0.033	0.026	0.032	0.030	0.077	0.041	0.000	0.058	0.013
PJL	0.042	0.040	0.048	0.050	0.048	0.043	0.077	0.019	0.058	0.000	0.071
STU	0.038	0.038	0.042	0.035	0.041	0.040	0.083	0.054	0.013	0.071	0.000

Supplemental Table 8) South Asian Euclidean distances between population centroids using South Asian ancestry-specific MDS1 and MDS2 coordinates.

bioRxiv preprint doi: https://doi.org/10.1101/2025.10.05.680541; this version posted October 7, 2025. The copyright holder for this preprint (which was not certified by peer review) is the author/funder, who has granted bioRxiv a license to display the preprint in perpetuity. It is made available under aCC-BY 4.0 International license.

	CDB	KRO	ORG	NCTB	Sulawesi	Java	Flores	Sumatra	Alor	Bali	Borneo	CHB	KHV	Kei	Lembata	Mentawai	Nias	Tanimbar
CDB	0.000	0.003	0.010	0.010	0.021	0.028	0.046	0.043	0.092	0.026	0.036	0.065	0.042	0.077	0.062	0.127	0.092	0.056
KRO	0.003	0.000	0.008	0.008	0.018	0.026	0.047	0.041	0.094	0.024	0.034	0.066	0.042	0.079	0.063	0.125	0.089	0.057
ORG	0.010	0.008	0.000	0.003	0.012	0.019	0.055	0.034	0.101	0.016	0.026	0.066	0.039	0.086	0.070	0.119	0.083	0.064
NCTB	0.010	0.008	0.003	0.000	0.011	0.019	0.054	0.033	0.101	0.017	0.026	0.068	0.041	0.086	0.070	0.117	0.082	0.064
Sulawesi	0.021	0.018	0.012	0.011	0.000	0.014	0.061	0.023	0.108	0.014	0.019	0.076	0.047	0.093	0.077	0.107	0.071	0.070
Java	0.028	0.026	0.019	0.019	0.014	0.000	0.073	0.019	0.120	0.003	0.009	0.069	0.037	0.105	0.089	0.104	0.068	0.082
Flores	0.046	0.047	0.055	0.054	0.061	0.073	0.000	0.084	0.047	0.071	0.079	0.096	0.083	0.032	0.016	0.160	0.127	0.010
Sumatra	0.043	0.041	0.034	0.033	0.023	0.019	0.084	0.000	0.131	0.021	0.011	0.086	0.054	0.115	0.100	0.085	0.049	0.093
Alor	0.092	0.094	0.101	0.101	0.108	0.120	0.047	0.131	0.000	0.118	0.126	0.130	0.125	0.016	0.031	0.204	0.172	0.038
Bali	0.026	0.024	0.016	0.017	0.014	0.003	0.071	0.021	0.118	0.000	0.012	0.067	0.036	0.103	0.087	0.107	0.071	0.081
Borneo	0.036	0.034	0.026	0.026	0.019	0.009	0.079	0.011	0.126	0.012	0.000	0.076	0.044	0.111	0.096	0.095	0.059	0.089
CHB	0.065	0.066	0.066	0.068	0.076	0.069	0.096	0.086	0.130	0.067	0.076	0.000	0.032	0.119	0.105	0.165	0.131	0.104
KHV	0.042	0.042	0.039	0.041	0.047	0.037	0.083	0.054	0.125	0.036	0.044	0.032	0.000	0.112	0.097	0.134	0.099	0.093
Kei	0.077	0.079	0.086	0.086	0.093	0.105	0.032	0.115	0.016	0.103	0.111	0.119	0.112	0.000	0.016	0.188	0.157	0.023
Lembata	0.062	0.063	0.070	0.070	0.077	0.089	0.016	0.100	0.031	0.087	0.096	0.105	0.097	0.016	0.000	0.175	0.143	0.009
Mentawai	0.127	0.125	0.119	0.117	0.107	0.104	0.160	0.085	0.204	0.107	0.095	0.165	0.134	0.188	0.175	0.000	0.036	0.167
Nias	0.092	0.089	0.083	0.082	0.071	0.068	0.127	0.049	0.172	0.071	0.059	0.131	0.099	0.157	0.143	0.036	0.000	0.135
Tanimbar	0.056	0.057	0.064	0.064	0.070	0.082	0.010	0.093	0.038	0.081	0.089	0.104	0.093	0.023	0.009	0.167	0.135	0.000

Supplemental Table 9) Southeast and East Asian distances. Euclidean distances between population centroids using Southeast and East Asian ancestry-specific MDS1 and MDS2 coordinates.

bioRxiv preprint doi: https://doi.org/10.1101/2025.10.05.680541; this version posted October 7, 2025. The copyright holder for this preprint (which was not certified by peer review) is the author/funder, who has granted bioRxiv a license to display the preprint in perpetuity. It is made available under aCC-BY 4.0 International license.

SPRUCE

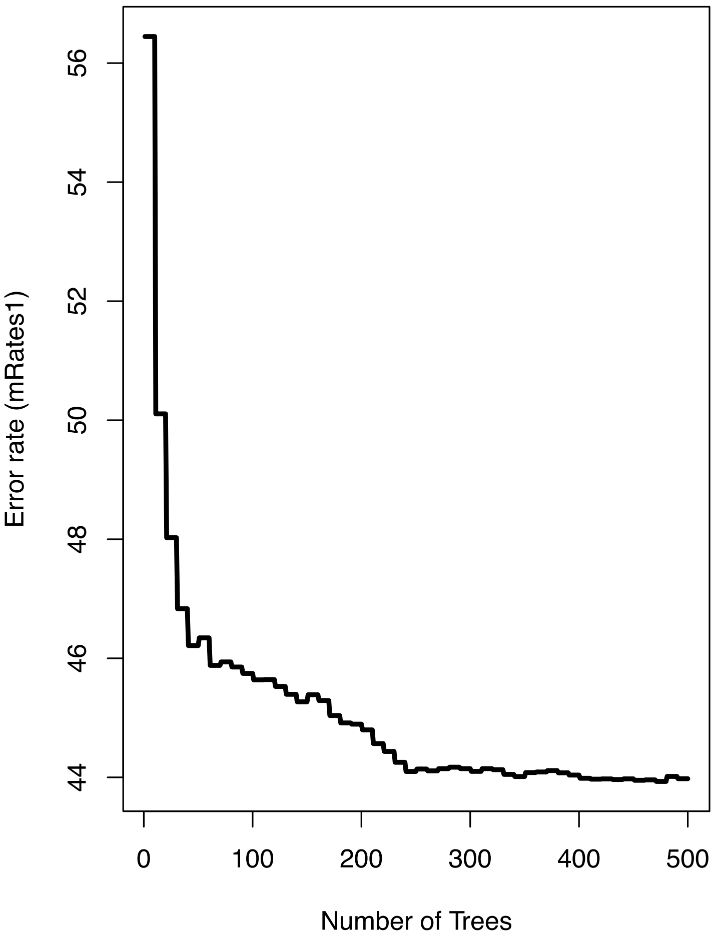

Supplemental Figure 23) Error rate plot showing the error rate decreasing as the number of trees in the random forest increases for IBD segments 2-6 cM representing 56-13 generations ago.

bioRxiv preprint doi: https://doi.org/10.1101/2025.10.05.680541; this version posted October 7, 2025. The copyright holder for this preprint (which was not certified by peer review) is the author/funder, who has granted bioRxiv a license to display the preprint in perpetuity. It is made available under aCC-BY 4.0 International license.

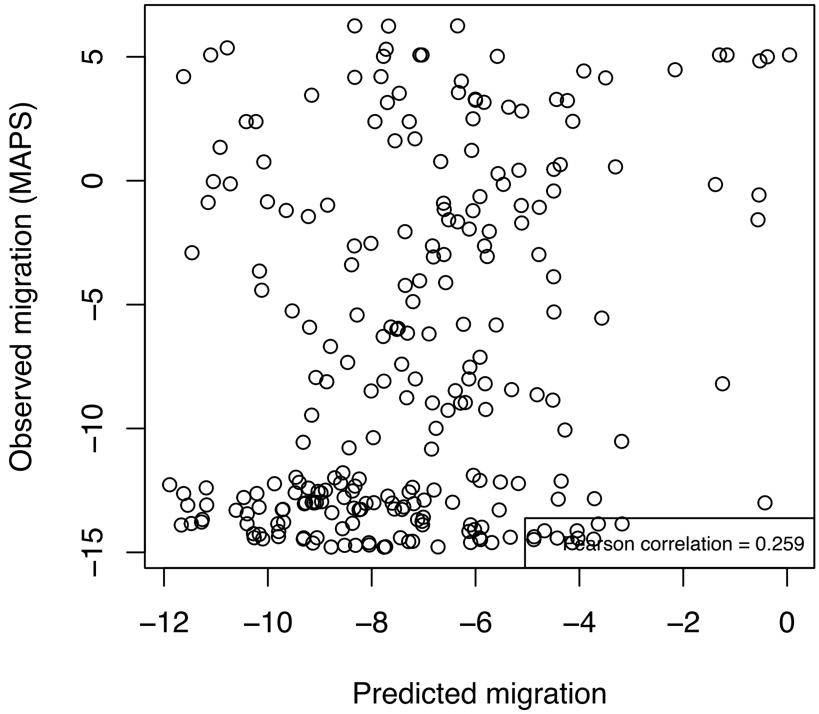

Supplemental Figure 24) Scatterplot showing predicted migration plotted against observed migration (Pearson correlations = 0.259) for IBD segments 2-6 cM/56-13 generations ago.

bioRxiv preprint doi: https://doi.org/10.1101/2025.10.05.680541; this version posted October 7, 2025. The copyright holder for this preprint (which was not certified by peer review) is the author/funder, who has granted bioRxiv a license to display the preprint in perpetuity. It is made available under aCC-BY 4.0 International license.

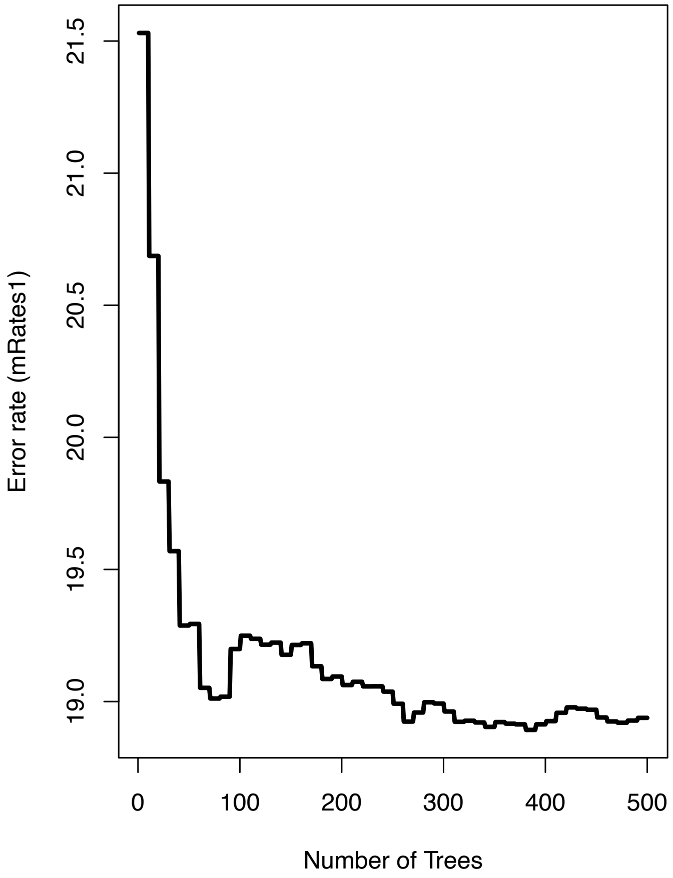

Supplemental Figure 25) Error rate plot showing the error rate decreasing as the number of trees in the random forest increases for IBD segments greater than 6 cM representing less than 13 generations ago.

bioRxiv preprint doi: https://doi.org/10.1101/2025.10.05.680541; this version posted October 7, 2025. The copyright holder for this preprint (which was not certified by peer review) is the author/funder, who has granted bioRxiv a license to display the preprint in perpetuity. It is made available under aCC-BY 4.0 International license.

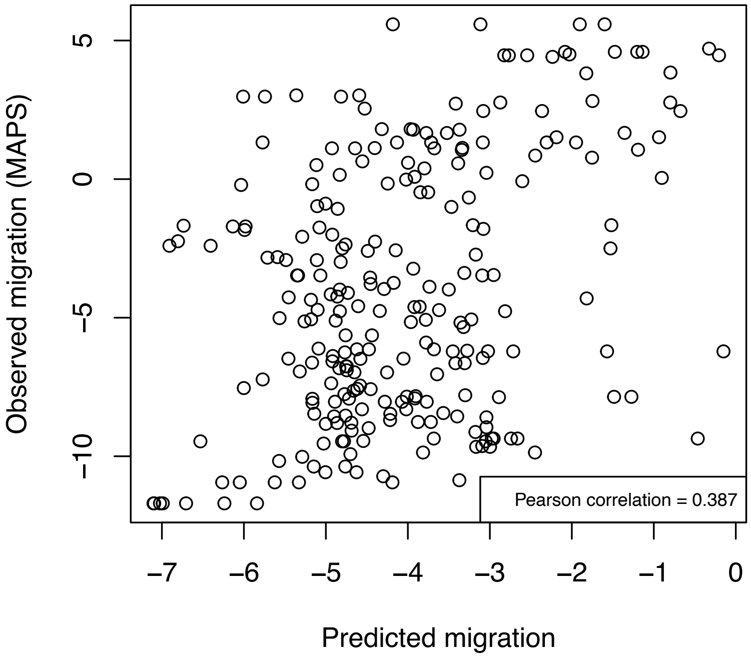

Supplemental Figure 26) Scatterplot showing predicted migration plotted against observed migration (Pearson correlations = 0.387) for IBD segments greater than 6 cM/ less than13 generations ago.

bioRxiv preprint doi: https://doi.org/10.1101/2025.10.05.680541; this version posted October 7, 2025. The copyright holder for this preprint (which was not certified by peer review) is the author/funder, who has granted bioRxiv a license to display the preprint in perpetuity. It is made available under aCC-BY 4.0 International license.

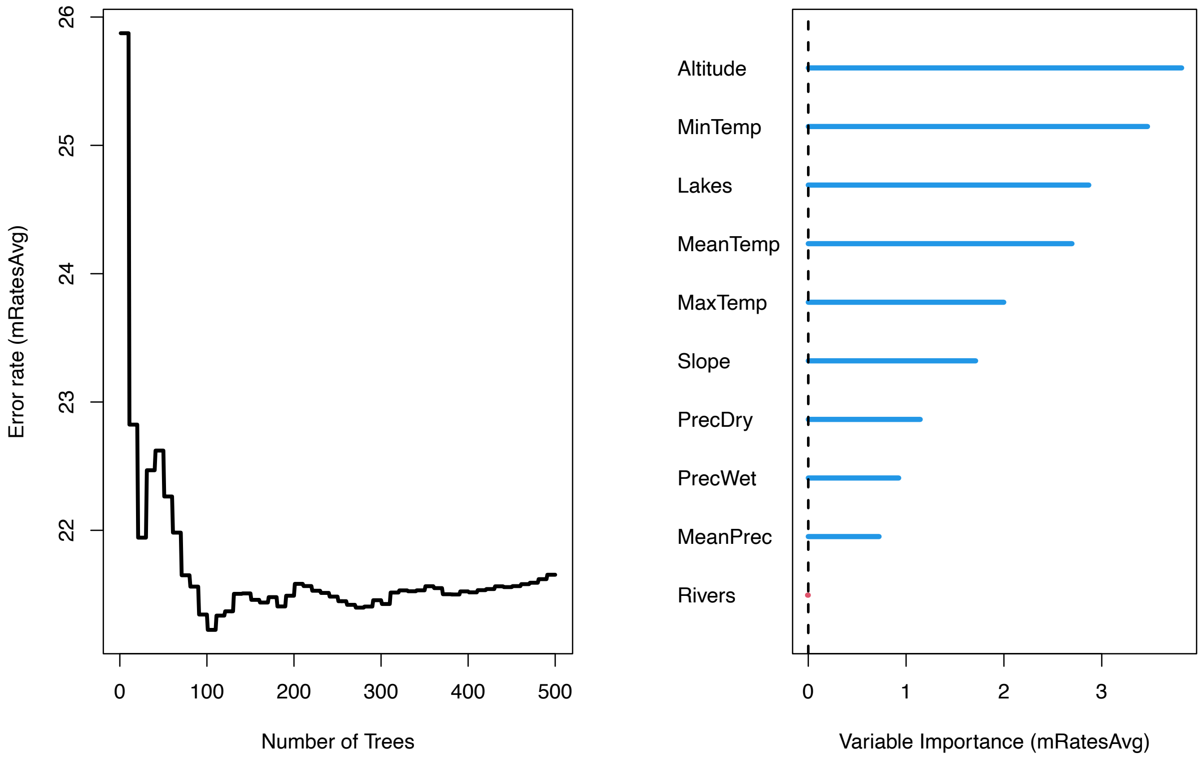

Supplemental Figure 27) SPRUCE Random forest importance table average across time periods (2-6cM and greater than 6cM), 56> generations ago showing altitude and minimum temperature during the coldest month being the most important variables explaining the variation in estimated migration rate.

bioRxiv preprint doi: https://doi.org/10.1101/2025.10.05.680541; this version posted October 7, 2025. The copyright holder for this preprint (which was not certified by peer review) is the author/funder, who has granted bioRxiv a license to display the preprint in perpetuity. It is made available under aCC-BY 4.0 International license.

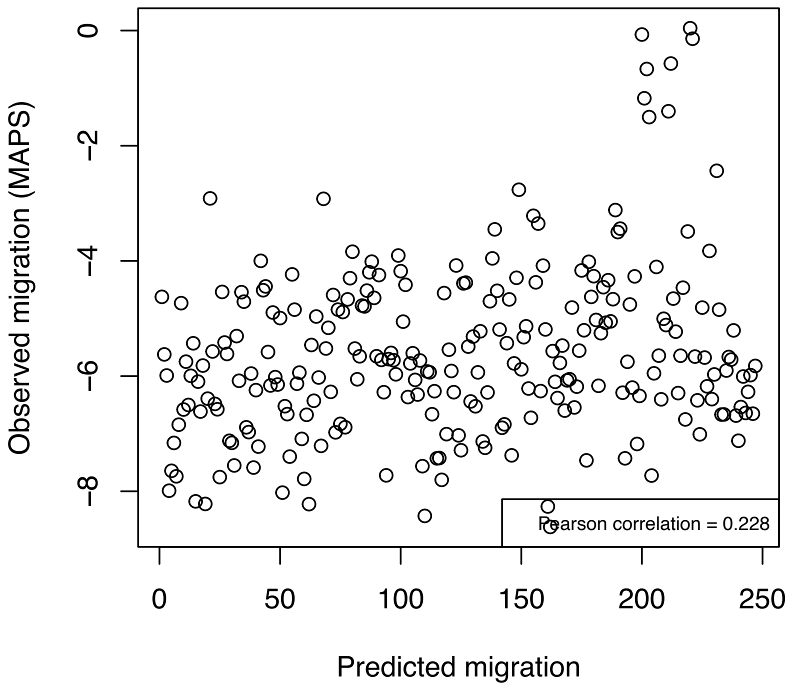

Supplemental Figure 28) Scatterplot showing predicted migration plotted against observed migration (Pearson correlations = 0.228) on average.

bioRxiv preprint doi: https://doi.org/10.1101/2025.10.05.680541; this version posted October 7, 2025. The copyright holder for this preprint (which was not certified by peer review) is the author/funder, who has granted bioRxiv a license to display the preprint in perpetuity. It is made available under aCC-BY 4.0 International license.

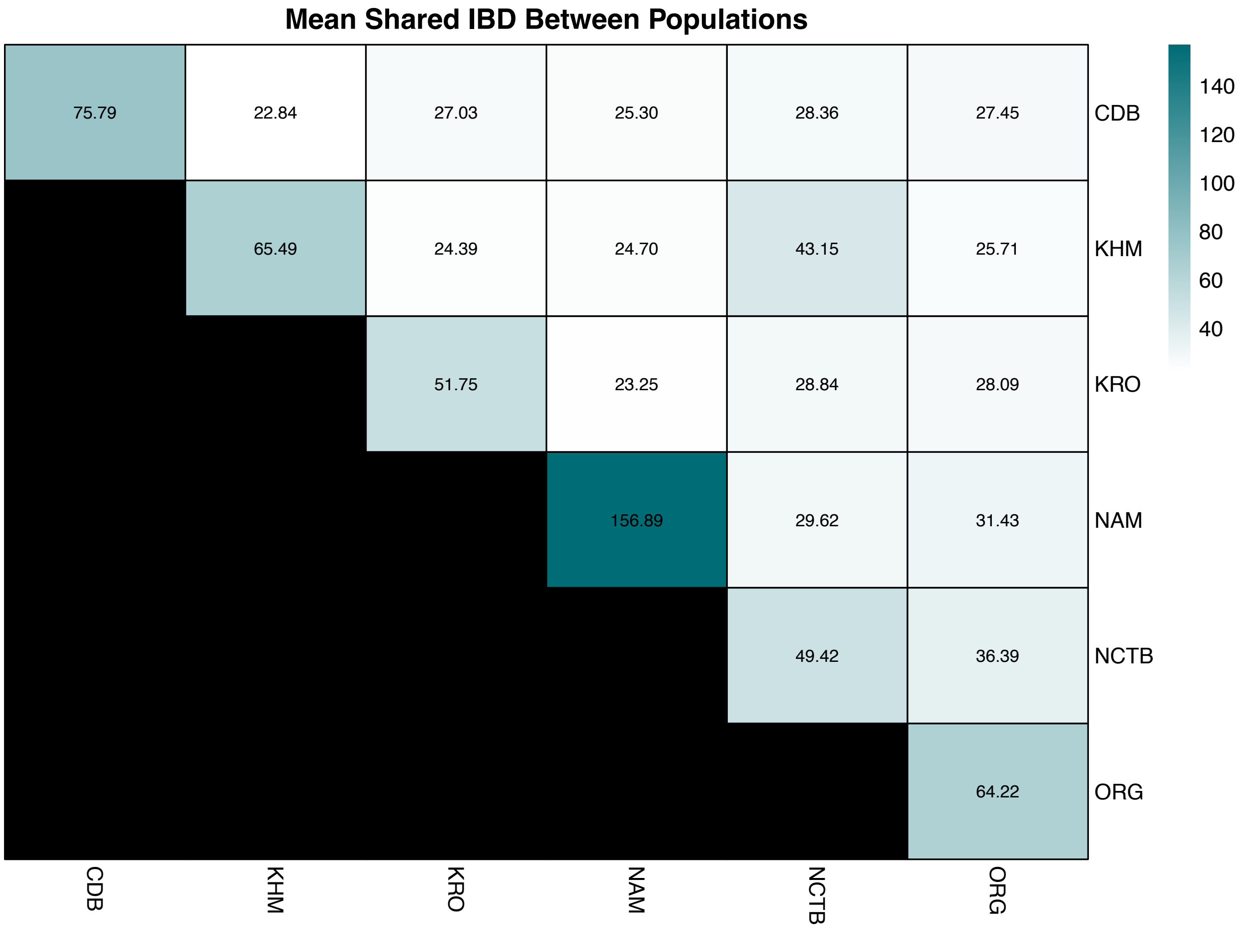

Supplemental Figure 29) Mean shared IBD length between and within Khoe-San and Khoe-San descendant communities.

bioRxiv preprint doi: https://doi.org/10.1101/2025.10.05.680541; this version posted October 7, 2025. The copyright holder for this preprint (which was not certified by peer review) is the author/funder, who has granted bioRxiv a license to display the preprint in perpetuity. It is made available under aCC-BY 4.0 International license.

References

1. Crawford, N.G., Kelly, D.E., Hansen, M.E.B., Beltrame, M.H., Fan, S., Bowman, S.L., Jewett, E., Ranciaro, A., Thompson, S., Lo, Y., et al. (2017). Loci associated with skin pigmentation identified in African populations. Sci New York N Y *358*, eaan8433. https://doi.org/10.1126/science.aan8433.

2. Fan, S., Spence, J.P., Feng, Y., Hansen, M.E.B., Terhorst, J., Beltrame, M.H., Ranciaro, A., Hirbo, J., Beggs, W., Thomas, N., et al. (2023). Whole-genome sequencing reveals a complex African population demographic history and signatures of local adaptation. Cell *186*, 923-939.e14. https://doi.org/10.1016/j.cell.2023.01.042.

3. Scrucca, L., Fop, M., Murphy, T.B., and Raftery, A.E. (2016). mclust 5: Clustering, Classification and Density Estimation Using Gaussian Finite Mixture Models. R J. *8*, 289–317.

4. McInnes, L., Healy, J., Saul, N., and Großberger, L. (2018). UMAP: Uniform Manifold Approximation and Projection. J. Open Source Softw. *3*, 861. https://doi.org/10.21105/joss.00861.

5. Malzer, C., and Baum, M. (2020). A Hybrid Approach To Hierarchical Density-based Cluster Selection. 2020 IEEE Int. Conf. Multisens. Fusion Integr. Intell. Syst. (MFI) *00*, 223–228. https://doi.org/10.1109/mfi49285.2020.9235263.

6. Patterson, N., Price, A.L., and Reich, D. (2006). Population Structure and Eigenanalysis. PLoS Genet. *2*, e190. https://doi.org/10.1371/journal.pgen.0020190.

7. Diaz-Papkovich, A., Zabad, S., Ben-Eghan, C., Anderson-Trocmé, L., Femerling, G., Nathan, V., Patel, J., and Gravel, S. (2023). Topological stratification of continuous genetic variation in large biobanks. bioRxiv, 2023.07.06.548007. https://doi.org/10.1101/2023.07.06.548007.

8. Reich, D., Thangaraj, K., Patterson, N., Price, A.L., and Singh, L. (2009). Reconstructing Indian population history. Nature *461*, 489–494. https://doi.org/10.1038/nature08365.

9. Metspalu, M., Mondal, M., and Chaubey, G. (2018). The genetic makings of South Asia. Curr. Opin. Genet. Dev. *53*, 128–133. https://doi.org/10.1016/j.gde.2018.09.003.

10. Kerdoncuff, E., Skov, L., Patterson, N., Zhao, W., Lueng, Y.Y., Schellenberg, G.D., Smith, J.A., Dey, S., Ganna, A., Dey, A., et al. (2024). 50,000 years of Evolutionary History of India: Insights from ~2,700 Whole Genome Sequences. bioRxiv, 2024.02.15.580575. https://doi.org/10.1101/2024.02.15.580575.

11. Moorjani, P., Thangaraj, K., Patterson, N., Lipson, M., Loh, P.-R., Govindaraj, P., Berger, B., Reich, D., and Singh, L. (2013). Genetic Evidence for Recent Population Mixture in India. Am. J. Hum. Genet. *93*, 422–438. https://doi.org/10.1016/j.ajhg.2013.07.006.
